# Human milk microbiota: origins, determinants, and roles in maternal-infant microbial transmission and infant microbiome assembly

**DOI:** 10.3389/fmicb.2026.1790225

**Published:** 2026-06-05

**Authors:** Guangyu Ma, Leixi Peng, Lang Qin, Rui Cai, Xi Tan, Rui Gao

**Affiliations:** 1 Reproductive Medical Center, Department of Obstetrics and Gynecology, West China Second University Hospital, Sichuan University, Chengdu, China; 2 Key Laboratory of Birth Defects and Related Diseases of Women and Children, Ministry of Education, West China Second University Hospital, Sichuan University, Chengdu, China; 3 Department of Obstetrics and Gynecology, West China Second University Hospital, Sichuan University, Chengdu, China

**Keywords:** breastfeeding, dietary modifications, human milk microbiota, infant healthy, infant microbiota, maternal-infant microbial transfer, prebiotics, probiotics

## Abstract

Human milk is a complex and dynamic biological fluid that provides essential nutrients and harbors a diverse, functional microbiota, playing a critical role in infant microbial colonization and early life development. The milk microbiota is derived from multiple maternal and environmental sources, including the maternal gut via the entero-mammary pathway, the mammary and skin microbiota, infant oral microbes through retrograde flow, and environmental exposures. Its composition is influenced by a range of factors, such as maternal metabolic and health status, diet, and antibiotic use, as well as delivery mode, lactation stage, infant characteristics, and geographic context. Human milk contributes to the establishment of oral, airway, and gut microbial communities by transferring key taxa such as *Bifidobacterium* and *Lactobacillus*, which are commonly detected in milk; however, direct evidence of specific strains establishing in the infant gut remains limited. Breastfeeding may partially compensate for microbiome deficits in cesarean-delivered, preterm, and antibiotic-exposed infants, supporting protection against infections, allergies, asthma, obesity, and other health outcomes. Translational strategies may help modulate the milk microbiota. These include maternal probiotic or prebiotic supplementation, dietary optimization, and approaches targeting microbiota or bioactive milk components. Such strategies offer feasible and cost-effective means to support healthy infant microbiome development. However, methodological constraints including low-biomass contamination, sequencing biases, and limited strain-level resolution remain significant challenges in accurately characterizing the human milk microbiota. Despite substantial advances, the relative contributions of distinct transmission routes, the persistence of maternal strains, and the efficacy of targeted maternal interventions remain incompletely understood. Addressing these gaps will be essential for refining strategies to promote healthy microbiome maturation and improve lifelong health outcomes.

## 1 Introduction

Human milk is widely recognized as the optimal source of infant nutrition. The World Health Organization (WHO) recommends exclusive breastfeeding for the first 6 months, followed by the introduction of complementary foods, with continued breastfeeding up to at least 2 years (World Health Organization, 2009; Victora et al., 2016). Human milk contains numerous bioactive components beyond macronutrients and micronutrients, including immune factors, hormones, growth factors, enzymes, and extracellular vesicles, which collectively support infant growth, immune development, and gut maturation (Boix-Amorós et al., 2019; Walsh et al., 2020; Zhang et al., 2022; Reyes et al., 2024; Çelik et al., 2024; Talbert and Townsend, 2025). In addition to these nutritional and immunological functions, human milk harbors a complex and dynamic microbiota that plays a critical role in shaping neonatal gut colonization, metabolome, and immune maturation (Vandenplas et al., 2020; Davis et al., 2022; Ma et al., 2023). Vaginal delivery allows the initial transfer of maternal bacteria to the infant, although this is a one-time event at birth and the number of bacteria transferred has not been precisely quantified in humans. In contrast, breastfeeding provides a daily source of microbes, with approximately 1 × 10^5^ to 1 × 10^7^ bacteria transferred per day, making it the second most significant contributor to early infant microbial exposure (Lyons et al., 2020; Selma-Royo et al., 2021).

Human milk was once considered sterile, but bacteria have been detected in the milk of healthy women using culture-dependent and viability-based methods. Culturomics and traditional cultivation studies have successfully isolated viable bacteria, including lactic acid bacteria and bifidobacteria, from human milk (Soto et al., 2014; Liu et al., 2020; El-Sayed et al., 2022). Additionally, viability-based sequencing using propidium monoazide (PMA) demonstrates that a subset of bacteria in fresh human milk represents viable cells rather than only DNA from non-viable organisms (Stinson et al., 2021). High-throughput sequencing and metagenomics have progressively unveiled the complex and diverse microbial communities inhabiting human milk (Hunt et al., 2011; Cabrera-Rubio et al., 2012; Jost et al., 2014; Ma et al., 2024). The milk microbiota originates from multiple maternal and environmental sources, including translocation from the maternal gut via the entero-mammary pathway, as well as contributions from the nipple and areolar skin and from the infant’s oral cavity through retrograde flow (Rodríguez, 2014; Kordy et al., 2020; Mantziari and Rautava, 2021; Dombrowska-Pali et al., 2024). Its composition undergoes dynamic, stage-specific changes throughout lactation, with distinct microbial profiles observed between colostrum and mature milk (Singh et al., 2023). Nevertheless, these patterns vary across cohorts, suggesting that milk microbiota development is highly individualized and shaped by both maternal and environmental factors (Wan et al., 2020).

Growing evidence demonstrates that the human milk microbiota may contribute to infant microbiome development and long-term health. Breast milk contributes to establishing the infant gut microbiota, guiding early microbial colonization, and shaping lifelong immune and metabolic trajectories (Pannaraj et al., 2017; Ma et al., 2024; Zhernakova et al., 2025), while also providing other bioactive components (e.g., HMOs, IgA) that influence these processes (Martín et al., 2007; Biesbroek et al., 2014a; Kaan et al., 2021). Breastfeeding has been associated with reduced neonatal morbidity and mortality, as well as lasting protection against allergies, asthma, obesity, diabetes, and inflammatory bowel disease (Horta et al., 2015; Klopp et al., 2017; Xu et al., 2017; Wan et al., 2020), though these effects are likely mediated by multiple milk components, not solely by the milk microbiota. Maternal diet, lifestyle, delivery mode, environmental exposures, and infant feeding practices represent key determinants of human milk microbiota composition, collectively influencing its diversity and functional potential (Moossavi et al., 2019; Padilha et al., 2019a; Wan et al., 2020; Cortes-Macías et al., 2021). Overall, these findings suggest that the milk microbiota is an important contributor to early microbial colonization and infant health. Its potential as a target for interventions, alongside other milk components, could help optimize microbiome development and reduce the risk of allergic, metabolic, and inflammatory disorders.

This review summarizes current knowledge on the human milk microbiota as a central determinant of infant microbiome development and health, with emphasis on its origins, maternal and environmental factors shaping its composition, and downstream implications for infant immunity and microbial colonization. By integrating microbial sources, milk components, and infant outcomes into a conceptual framework, this review provides a systematic perspective to guide future research and inform potential strategies for targeted modulation of maternal milk and infant microbiomes to support optimal growth and long-term health.

## 2 Literature search strategy

Relevant literature was identified through searches of PubMed and Web of Science databases for studies published up to 2025. Search terms included combinations of “human milk microbiota,” “breast milk microbiome,” “milk mycobiome,” “maternal-infant microbial transmission,” “infant microbiota,” “breastfeeding,” “probiotics,” “prebiotics,” and “microbial origins.” Human studies, animal studies, and sequencing-based investigations relevant to the composition, origins, transmission, and functional implications of the human milk microbiota were considered. Additional references were identified through manual screening of cited literature.

## 3 Origins of the human milk microbiota

Human milk harbors a diverse microbiome, including bacteria, fungi, and potentially viruses, which may influence infant microbial colonization and health (Consales et al., 2022; Guo et al., 2025). Most studies have focused on bacterial communities, although increasing evidence suggests that fungal components of human milk (the milk mycobiome) also contribute to infant immune development and gut ecology (Boix-Amorós et al., 2017; Ward et al., 2017). Characterization of the milk microbiome relies primarily on culture-independent approaches, including 16S rRNA gene sequencing, ITS sequencing, and shotgun metagenomics (Ruiz et al., 2019b; Couvillion et al., 2023). Despite methodological challenges related to low microbial biomass and contamination susceptibility, recent studies have advanced understanding of the human milk microbiome and its potential origins (Stinson et al., 2021; Ferretti et al., 2025).

Current evidence suggests that human milk microbial communities originate from multiple maternal and infant-associated sources, including the maternal gut, infant oral cavity, nipple and areolar skin, mammary tissue, and environmental exposures. Table 1 summarizes key studies investigating the origins and transmission routes of these microbes.

**TABLE 1:** Key studies investigating the sources and transmission routes of human milk microbiota.

Study (year)	Source of human milk microbiota	Experimental protocol	Research findings
Jiménez et al. (2008)	Maternal gut (*Lactobacillus salivarius*, *Lactobacillus gasseri*)	Oral administration of *Lactobacillus* strains to 10 lactating women; milk collected at day 0 and 30; bacteria identified by culture and molecular methods.	Orally administered *Lactobacillus* detected in milk of 6/10 women, supporting maternal gut origin via entero-mammary pathway.
Donnet-Hughes et al. (2010)	Maternal gut (bacteria translocated via PBMC/DC to breast milk)	Human milk, maternal blood, and infant feces collected; milk cells and PBMC analyzed; conventional mice studied for bacterial translocation.	Bacterial DNA and immune cells detected in milk and PBMC; shared taxa included *Bifidobacterium*, *Streptococcus thermophilus*, *Staphylococcus epidermidis*; mouse data supported increased bacterial translocation during lactation.
Jost et al. (2014)	Maternal gut (*Bifidobacterium*, *Bacteroides*, *Parabacteroides*, *Clostridia*)	Maternal feces, milk, and neonatal feces from 7 mother-infant pairs at 3 time points; sequencing and culture.	Gut-associated anaerobes detected in milk and infant feces; *Bifidobacterium breve* and butyrate-producing Clostridia shared across maternal gut, milk, and infant; supports vertical transfer via entero-mammary pathway.
Urbaniak et al. (2014a)	Breast tissue (*Proteobacteria*, *Firmicutes*, *Actinobacteria*, *Bacteroidetes*, others)	Breast tissue from 81 women (healthy and cancer) analyzed by 16S rRNA sequencing; Canadian samples cultured on Columbia blood agar.	Diverse bacterial community detected in all samples; *Proteobacteria* most abundant, followed by *Firmicutes*; viable bacteria confirmed (*Bacillus*, *Micrococcus*, *Propionibacterium*, *Staphylococcus*, *Streptococcus*); breast tissue may serve as intrinsic source for milk microbiota
Milani et al. (2015)	Maternal gut (*Bifidobacterium breve*, *Bifidobacterium longum subspecies longum*)	Four mother-infant pairs; fecal and milk samples at 3 and 6 months; strain isolation, ITS profiling, PCR, genome sequencing.	Identical *Bifidobacterium* strains detected in maternal gut, milk, and infant feces, confirming vertical transmission
Hieken et al. (2016)	Mammary gland tissue	Sterile breast tissue from women with benign and malignant disease; paired skin tissue and buccal swabs; 16S rDNA sequencing.	Breast tissue has distinct microbiome from skin/buccal; greater species richness; rare genera detected (*Fusobacterium*, *Atopobium*, *Lactobacillus*); supports intrinsic milk microbiota source.
Duranti et al. (2017)	Maternal gut to breast milk to infant gut (*Bifidobacterium* strains)	25 mother-infant pairs; maternal feces at delivery, milk at 7 and 30 days, infant feces at 7 days and 1 month; ITS profiling, cultivation, genome sequencing, qPCR, metagenomics.	Identical *Bifidobacterium* strains and bifidophages shared across gut, milk, and infant feces; supports entero-mammary pathway.
de Andrés et al. (2017)	Maternal gut (*Lactococcus lactis*, *Lactobacillus salivarius*)	Lux-labeled *Lactobacillus salivarius* PS2 and *Lactococcus lactis* MG1614 orally administered to pregnant mice; detection in mammary tissue and milk.	Labeled bacteria translocated from gut to mammary gland and milk, confirming entero-mammary pathway.
Kordy et al. (2020)	Maternal areolar skin, infant oral cavity (retrograde transfer); maternal gut (entero-mammary pathway)	20 mother-infant pairs (15 latched, 5 never-latched); milk, maternal oral/areolar/vaginal/rectal, infant oral/stool samples; metagenomic strain comparison.	Milk microbiota mainly derived from areolar skin (∼46%) and infant oral cavity (∼26%); one C-section dyad showed identical *Bifidobacterium breve* in maternal rectum, milk, and infant stool, supporting gut-to-milk transfer.
Laursen et al. (2021)	Maternal skin, infant oral cavity, maternal gut and oral cavity	Foremilk and hindmilk collected before and after breastfeeding; infant feces at 5 and 9 months.	Hindmilk had higher bacterial load and more oral-associated bacteria; 33% (5 months) and 23% (9 months) infant fecal ASVs shared with milk; bidirectional transfer (*Streptococcus*, *Veillonella*, *Rothia*) from infant oral cavity to milk; maternal gut/oral contribution also observed.
Juárez-Castelán et al. (2022)	Maternal gut	50 Mother-neonate pairs; colostrum, maternal and neonatal rectal swabs; SourceTracker analysis.	∼21% Of colostrum microbiota shared with maternal gut; dominant genera included *Staphylococcus*, *Streptococcus*, *Escherichia*, *Microbacterium*, *Prevotella*.
Qi et al. (2022)	Maternal gut (*Faecalibacterium*, *Blautia*, *Lachnoclostridium*, *Streptococcus salivarius*, *Bifidobacterium longum*, *Lactobacillus gasseri*)	Colostrum, transitional, and mature milk from 19 mother-infant dyads; bacterial ASVs compared between maternal gut, milk, and infant gut.	Gut-associated anaerobes and probiotic taxa detected in milk and infant gut; transfer highest during colostrum stage, supporting entero-mammary pathway.
Xu et al. (2025)	Infant oral cavity (retrograde), maternal skin, possible maternal gut	Aseptic milk collection via hand expression after nipple disinfection; taxonomic assignment using SILVA v132.	Dominant oral/skin-associated genera (*Streptococcus*, *Cutibacterium*, *Staphylococcus*); oral commensals increased during breastfeeding; *Bifidobacterium* detected in 1/3 samples, suggesting possible maternal gut contribution.

ASV, amplicon sequence variant; DC, dendritic cell; ITS, internal transcribed spacer region; PBMC, peripheral blood mononuclear cell; qPCR, quantitative polymerase chain reaction.

### 3.1 Entero-mammary pathway: maternal gut origin

**FIGURE 1:**
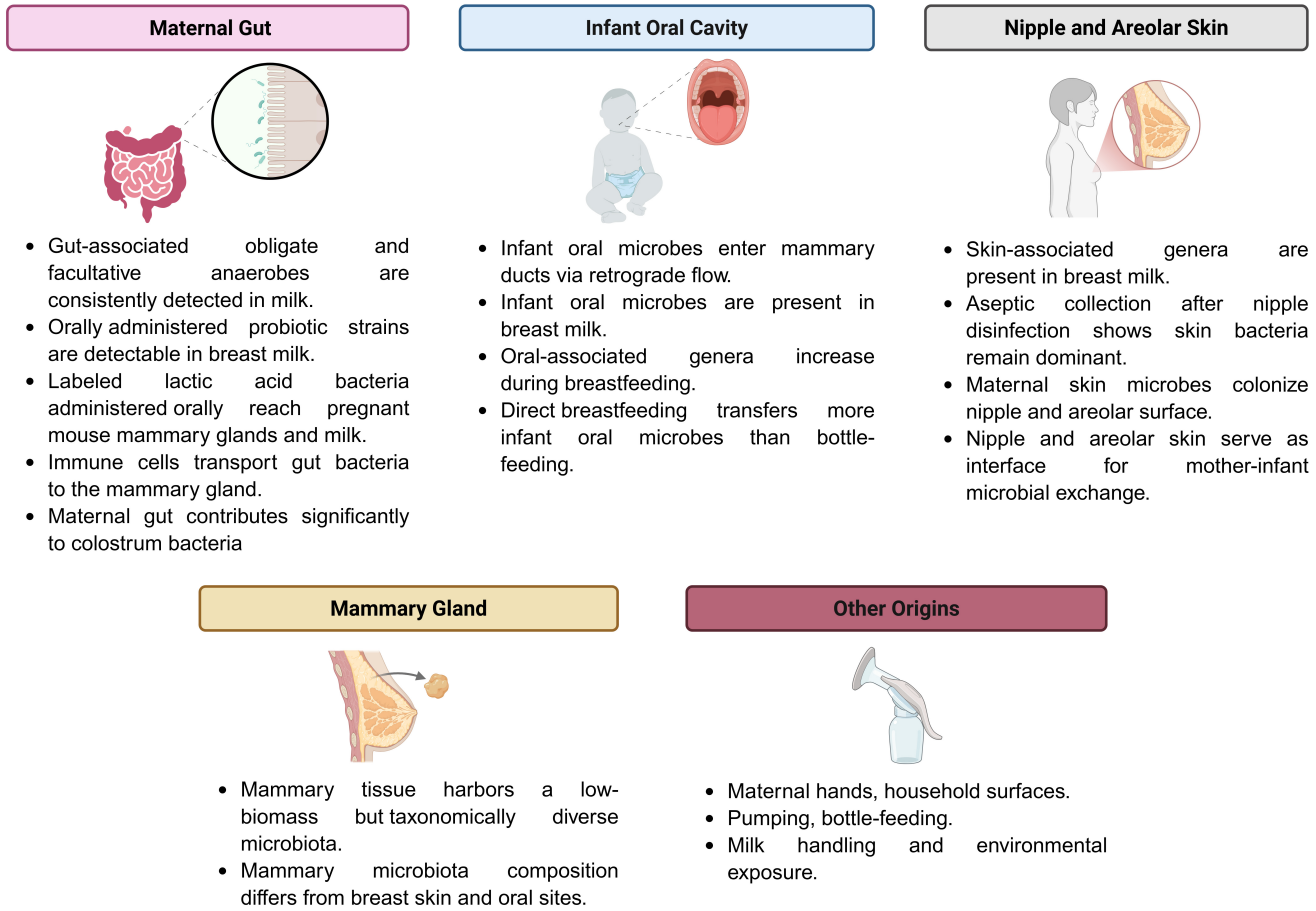
Proposed sources and routes of microbial transfer to human milk. The human milk microbiota originates from multiple maternal, infant oral, and environmental sources. Maternal gut bacteria, including probiotics, can reach the mammary gland via the entero-mammary pathway, potentially mediated by immune cells, and contribute substantially to colostrum bacteria. During breastfeeding, infant oral microbes may enter mammary ducts through retrograde flow, with oral-associated genera becoming more abundant in milk; direct breastfeeding transfers more microbes than bottle-feeding. Skin-associated bacteria are consistently detected in milk even after aseptic collection, with the nipple and areolar skin serving as a key interface for maternal-infant microbial exchange. Mammary gland tissue harbors a low-biomass yet taxonomically diverse microbiota, distinct from skin and oral sites, representing a potential intrinsic source. Environmental inputs, including maternal hands, household surfaces, milk handling, and pumping, further shape the complex microbial ecosystem of human milk.

The human milk microbiota is partly established through the entero-mammary pathway, in which maternal gut bacteria translocate to the mammary gland during pregnancy and lactation (Figure 1). Gut-associated taxa, including obligate anaerobes (*Bacteroides*, *Bifidobacterium*), facultative anaerobes (*Lactobacillus*), and *Clostridia* (e.g., *Faecalibacterium*, *Roseburia*), are consistently detected in milk, suggesting a potential internal maternal source (Jost et al., 2013; Rodríguez, 2014; Murphy et al., 2017; Corona-Cervantes et al., 2020).

Multiple lines of evidence support this mechanism. Probiotic strains such as *Lactobacillus salivarius* and *Lactobacillus gasseri*, administered orally, have been recovered from milk (Jiménez et al., 2008). Studies integrating culture-dependent and culture-independent methods demonstrate that maternal gut, milk, and infant feces share anaerobic taxa including *Bifidobacterium*, *Bacteroides*, *Parabacteroides*, and several *Clostridia* species (Jost et al., 2014). Strain-level analyses using culture-dependent isolation, internal transcribed spacer (ITS) profiling, quantitative PCR, and whole-genome sequencing show that identical *Bifidobacterium* species (*B. breve*, *B. longum*, *B. bifidum*) persist across the maternal gut-milk-infant triad for months (Milani et al., 2015; Duranti et al., 2017). These strains utilize human milk oligosaccharides (HMOs) and play a key role in infant gut development, supporting the possibility of milk-mediated vertical transmission.

Experimental data further reinforce gut-to-mammary translocation. In pregnant mice, Lux-labeled *Lactobacillus salivarius* PS2 and *Lactococcus lactis* MG1614, administered orally, appeared in mesenteric lymph nodes, mammary tissue, and milk; these effects were not observed in non-pregnant animals (de Andrés et al., 2017). In humans, sequencing-based source-tracking studies estimate that approximately 20–25% of colostrum bacteria may originate from the maternal gut, dominated by *Faecalibacterium*, *Blautia*, *Bifidobacterium*, and *Lactobacillus* during early lactation (Juárez-Castelán et al., 2022; Qi et al., 2022). However, given the low-biomass nature of colostrum and limitations of sequencing methods, these studies cannot definitively distinguish active bacterial translocation from passive DNA transfer or potential contamination. Therefore, while these data are consistent with a role for the entero-mammary pathway, further human studies employing strain-level tracking and functional assays are required to confirm active maternal gut-to-milk microbial transfer.

Mechanistically, hormonal changes in late pregnancy and early lactation increase intestinal permeability by modulating tight junctions (Kerr et al., 2015; Zhou et al., 2019; Ribeiro et al., 2023). Certain maternal gut bacteria cross the intestinal barrier, migrate via mucosal dendritic cells to Peyer’s patches, and are transported by lymphocytes to mesenteric lymph nodes (Perez et al., 2007; Civardi et al., 2013; Rodríguez, 2014). During lactation, these gut-associated lymphoid cells carry bacteria through lymphatic and systemic circulation to the mammary gland, where they are released into milk. However, the specific bacterial taxa involved remain largely unknown, and direct evidence in humans is currently lacking. Observations in lactating mice show increased bacterial translocation from the gut and higher numbers of dendritic cells carrying bacteria in mammary tissue (Perez et al., 2007; Donnet-Hughes et al., 2010). Occasional detection of bacterial DNA in maternal blood and breast milk cells has also been reported (Perez et al., 2007), although direct evidence demonstrating viable strain transfer to the infant gut remains lacking.

Short-chain fatty acids (SCFAs) (acetate, butyrate, formate) are detectable in human milk (Jiang et al., 2016; Prentice et al., 2019). While traditionally considered gut-derived metabolites, it remains unresolved whether these are produced by resident milk microbes, translocated gut bacteria, or transported directly from the maternal gut.

The mammary gland microenvironment actively shapes microbial colonization. Mammary epithelial cells and resident macrophages form an immune barrier, secreting antimicrobial peptides that selectively constrain bacterial persistence (Tunzi et al., 2000; Murakami et al., 2005). Milk nutrients, including lactose and HMOs, provide substrates supporting the growth and metabolic activity of specific taxa (Lawson et al., 2020; Lugli et al., 2020). Microbial interactions within milk, including competition and cooperation, further determine which bacteria establish and persist. These ecological dynamics are modulated by maternal diet, probiotic intake, and other environmental factors, underscoring the dynamic nature of early milk microbiota assembly.

Many gut-derived bacteria are obligate anaerobes, adapted to low-oxygen environments. Although the relatively oxygenated mammary gland may limit their long-term proliferation, microenvironments within milk, such as fat globules, epithelial microvesicles, or mucin-rich fractions, may serve as protective niches, allowing transient survival sufficient for transfer to the infant gut. Facultative anaerobes, such as *Lactobacillus*, tolerate oxygen better and may persist more actively in milk.

Despite strong evidence for the entero-mammary pathway, challenges remain. Methodological limitations, inter-individual and temporal variability, and alternative contributions, including retrograde flow and environmental exposure, complicate interpretation. Addressing these gaps requires strain-level tracking, longitudinal studies, and functional analyses.

Overall, current evidence suggests a dual mechanism for human milk microbiota: the entero-mammary pathway likely delivers gut-derived microbes, while the mammary microenvironment may selectively influence microbial survival and growth via immune, nutritional, and ecological filters. Together, these processes may contribute to maternal-infant microbial transfer and potentially influence early gut colonization and neonatal immune development.

### 3.2 Retrograde flow from the infant oral cavity

Retrograde flow from the infant oral cavity represents a physiologically plausible contributor to human milk microbiota (Figure 1). Ultrasound and pressure sensor studies, though not directly measuring microbial backflow, show synchronized intra-oral vacuum and milk flow during suckling, suggesting that cyclic pressure fluctuations may facilitate microbial transfer into mammary ducts (Geddes et al., 2008). Early lactation physiology proposed that microorganisms from the infant’s oral cavity or pre-milk secretions could enter the ducts during suckling, a concept formalized as retrograde inoculation (Latuga et al., 2014; Moossavi and Azad, n.d.).

Studies of mother-infant pairs indicate that milk collected during direct breastfeeding contains higher abundances of oral-associated genera, such as *Streptococcus*, *Veillonella*, and *Rothia*, consistent with infant-to-milk microbial transfer (Kordy et al., 2020). Longitudinal sequencing further reveals enrichment of oral-associated genera, including *Streptococcus*, *Veillonella*, and *Rothia*, after nursing, with species such as *Streptococcus salivarius*, *Streptococcus lactarius*, *Rothia mucilaginosa*, and *Granulicatella adiacens* increasing over time (Xu et al., 2025; Moossavi and Azad, n.d.). Hindmilk contains higher bacterial loads and more oral-associated taxa than foremilk, and 23–33% of infant fecal ASVs are shared with milk at 5–9 months, highlighting bidirectional maternal-infant microbial exchange (Laursen et al., 2021).

Despite these findings, most studies cannot definitively resolve the directionality of transfer (milk-to-oral versus oral-to-milk). Quantitative assessment of retrograde flow, including the proportion of milk microbiota derived from the infant oral cavity, remains largely unexplored. Physiological drivers, such as intraductal pressure fluctuations, lactation dynamics, and breast anatomy, are still largely speculative. Advanced imaging and animal experiments using labeled strains have begun to investigate these processes, but their precise contribution to retrograde microbial transfer, and its impact on milk composition, maternal breast health, and infant gut colonization, remains to be fully characterized.

Taken together, sequencing evidence, physiological plausibility, and emerging mechanistic data support infant-to-milk microbial transfer via retrograde flow, positioning this pathway as an important, yet incompletely characterized, contributor to human milk microbiota.

### 3.3 Contribution from nipple and areolar skin

In addition to bacteria originating from the maternal gut, both the nipple and the areolar skin contribute substantially to the human milk microbiota (Figure 1). These regions harbor skin-associated genera, including *Staphylococcus*, *Corynebacterium*, and *Cutibacterium*, which are also detected in milk, suggesting microbial transfer from maternal skin (Kordy et al., 2020; Xu et al., 2025). High-throughput sequencing and metagenomic analyses indicate that up to 46% of milk bacteria originate specifically from the areolar skin (Kordy et al., 2020). Even after nipple disinfection, skin-associated genera such as *Staphylococcus* and *Cutibacterium* remain prevalent in milk, highlighting the important role of maternal skin, particularly the areolar region, as a microbial reservoir (Xu et al., 2025).

Breastfeeding facilitates bidirectional microbial transfer, with the nipple and areolar skin serving as important sources of maternal skin bacteria and an interface for mother-infant microbial exchange. Hindmilk collected after direct nursing exhibits higher bacterial loads and increased abundance of oral-associated genera, consistent with retrograde flow from the infant’s oral cavity, while also reflecting contributions from maternal skin microbiota (Laursen et al., 2021; Xu et al., 2025).

Factors modulating skin-derived contributions include maternal skin hygiene, breastfeeding method (direct vs. pumped), and nipple condition. Overall, these findings indicate that the nipple and areolar skin serve as an interface for maternal and infant microbial exchange, complementing contributions from maternal gut and infant oral microbiota.

### 3.4 Mammary gland tissue microbiota

Non-lactating human mammary tissue appears to harbor a low-biomass but taxonomically diverse microbial signal, which could potentially contribute to milk composition during lactation, supporting the hypothesis that mammary tissue may host a distinct microbiota with possible implications for breast health (Figure 1). Analyses of sterilely collected breast tissue from women in Canada and Ireland suggest a predominance of *Proteobacteria*, followed by *Firmicutes*, which is distinct from the microbiota of breast skin or the oral cavity (Urbaniak et al., 2014a). However, most studies do not specify the exact anatomical subsite sampled (e.g., adipose tissue, ductal glands, or lobules), which may introduce variability and act as a potential confounding factor. The differences from skin or oral microbiota may reflect tissue-specific microenvironments, including composition, oxygen availability, and immune or metabolic factors (Thaiss et al., 2016; Hieken et al., 2022; Wong et al., 2025). However, these observations are based on low-biomass samples detecting mainly bacterial DNA, with no direct evidence of viable bacteria or gut-to-mammary translocation, leaving the contribution of tissue-resident microbes to lactational milk microbiota uncertain.

In breast cancer patients, genera such as *Fusobacterium* and *Atopobium* were enriched, suggesting potential links to pathology (Hieken et al., 2016). Comparative studies across healthy tissue, precancerous lesions, and tumors indicate that compositional shifts and metabolic reprogramming can occur even at the precancerous stage (Hoskinson et al., 2022). During lactation, it is possible that active milk secretion allows some tissue-associated microbes to enter milk and influence its composition; however, direct evidence from human studies is limited.

Overall, these studies provide preliminary insights into a potential mammary tissue microbiota. Given methodological limitations, low-biomass sampling, and the lack of precise anatomical localization, direct evidence for live bacterial transfer into milk is lacking. Future research using culture-based and strain-level approaches in clearly defined mammary subsites of lactating women will be important to clarify their existence and functional relevance.

### 3.5 Other environmental and exogenous origins

In addition to maternal gut, infant oral cavity, nipple and areolar skin, and mammary tissue, human milk microbiota may also be influenced by environmental and exogenous sources (Figure 1). Milk expression via pumps can introduce microbes from the pump equipment and maternal skin, whereas bottle feeding may further expose milk to infant oral microbes and environmental surfaces. Other potential sources include maternal hands, household surfaces, and strains from probiotics or functional foods (Boo et al., 2001; Gardner et al., 2015; Reyes et al., 2021, 2025; Moossavi and Azad, n.d.). Although these exogenous microbes are often transient or low in abundance, they can still contribute to early infant gut colonization and influence the establishment of milk-associated microbial communities.

## 4 Factors affecting the human milk microbiota

**FIGURE 2:**
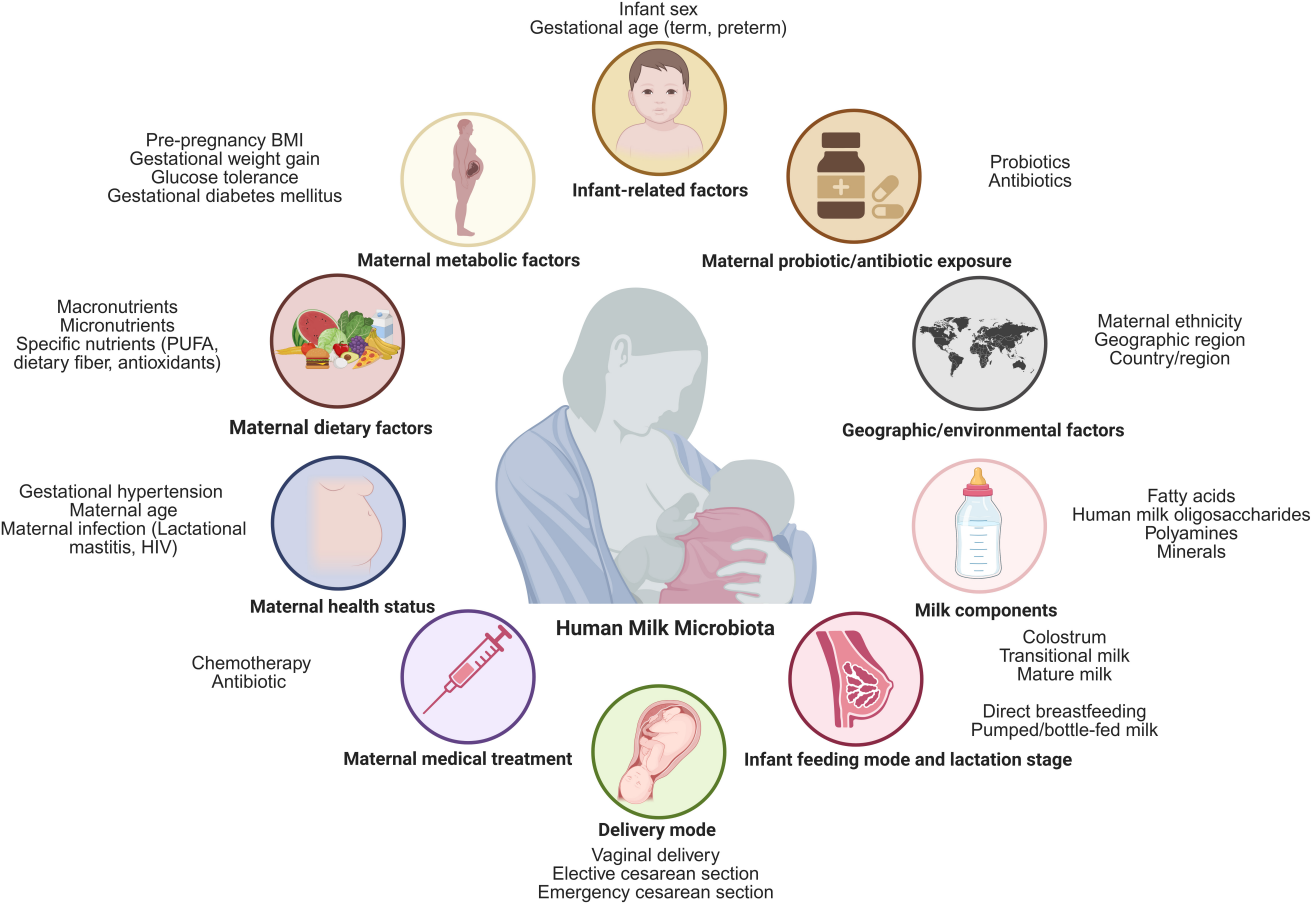
Factors affecting human milk microbiota composition. Maternal, infant, and environmental factors influence milk microbiota through metabolic, immune, and microbial pathways. Key modulators include maternal metabolic status, diet, antibiotic exposure, delivery mode, lactation stage, milk components, and infant feeding mode. These factors act directly on the mammary gland or indirectly via the entero-mammary pathway, collectively shaping microbial diversity and the transfer of beneficial microbes to the infant gut.

The composition of human milk microbiota is influenced by multiple factors, which can directly or indirectly shape infant microbial colonization, highlighting potential strategies to improve infant health by modulating milk microbiota through maternal diet, probiotics, or lifestyle interventions (Figure 2).

### 4.1 Maternal factors

Maternal characteristics are among the strongest determinants of human milk microbiota, influencing both microbial diversity and the abundance of taxa relevant to infant gut colonization and immune modulation (Figure 2). Overall, available evidence suggests that maternal metabolic status, dietary intake, and health conditions act in an interconnected rather than independent manner in shaping milk microbial profiles.

Maternal metabolic status consistently emerges as a key determinant of milk microbiota composition. In general, metabolic dysregulation-including obesity and excessive gestational weight gain-is associated with reduced colostrum and early milk diversity, along with shifts in key taxa, including increased *Lactobacillus*, *Staphylococcus*, and *Bacteroidetes*, and decreased *Bifidobacterium* and *Bacteroides* (Cabrera-Rubio et al., 2012; Williams et al., 2017; LeMay-Nedjelski et al., 2020). Similar patterns are observed in gestational diabetes mellitus (GDM), although findings are less consistent. While some studies report no significant differences in diversity with dominance of *Streptococcus* and *Staphylococcus* (Rold et al., 2024), others describe reduced abundances of *Sutterella*, *Serratia*, and *Lactococcus* (Valencia-Castillo et al., 2025), or conversely increased diversity in colostrum with enrichment of *Staphylococcus*, *Corynebacterium 1*, *Anaerococcus*, *Prevotella*, and *Rhodobacteraceae* (Gámez-Valdez et al., 2021), suggesting heterogeneity likely related to differences in metabolic severity and gut–mammary axis alterations.

Beyond individual diagnoses, interactions between body mass index (BMI), glucose tolerance, and postpartum adiposity further modulate microbial composition, as reflected by increased Gemella in metabolically dysregulated mothers (LeMay-Nedjelski et al., 2020). In contrast, metabolically healthier phenotypes appear to be associated with more favorable milk microbial profiles, as mothers with normal pre-gestational BMI who exclusively breastfed exhibited higher alpha-diversity and greater abundance of *Bifidobacterium* compared with those with mixed feeding practices or altered BMI (Cortés-Macías et al., 2021). Collectively, these findings indicate that metabolic status and feeding practices jointly contribute to shaping milk microbiota composition, particularly in terms of diversity and enrichment of beneficial taxa.

Maternal diet is associated with variations in milk microbiota, likely via substrate availability and modulation of gut ecology. Observational studies consistently report associations between dietary components and milk bacterial composition. Macronutrients such as protein correlate with *Gemella*, whereas monounsaturated fatty acids (MUFA) and saturated fatty acids (SFA) are inversely associated with Corynebacterium (Williams et al., 2017). Micronutrients and bioactive compounds, including riboflavin, calcium, vitamins D and E, chromium, zinc, and vitamin C, are also linked to specific taxa, while polyunsaturated fatty acids (PUFAs) and linoleic acid are associated with higher relative abundance of *Bifidobacterium* (Williams et al., 2017; Padilha et al., 2019a; Bzikowska-Jura et al., 2025). These findings support a dual mechanism in which diet may influence milk microbiota directly via milk biochemistry and indirectly through the entero-mammary pathway.

Maternal health status and clinical interventions further contribute to inter-individual variability in milk microbiota. Hypertensive disorders of pregnancy, such as gestational prehypertension, are associated with reduced microbial diversity and decreased *Lactobacillus* abundance, potentially reflecting systemic inflammatory or immune-mediated effects (Wan et al., 2020). Cytotoxic chemotherapy markedly alters milk microbial composition, leading to depletion of beneficial taxa such as *Bifidobacterium* and enrichment of opportunistic organisms, likely reflecting systemic disruption of host-microbiome homeostasis (Urbaniak et al., 2014b). Infectious and inflammatory conditions such as mastitis are characterized by reduced community diversity and dominance of Staphylococcus aureus or *Staphylococcus* epidermidis, contrasting with the more diverse bacterial, fungal, and protozoal communities observed in healthy milk (Jiménez et al., 2015). Similarly, HIV infection has been associated with increased microbial diversity and elevated *Lactobacillus* abundance, potentially reflecting immune-mediated alterations in epithelial and microbial niches (González et al., 2013). Mechanistic studies are still needed to clarify causal pathways linking maternal health conditions and therapeutic interventions to milk microbiota alterations.

Overall, maternal metabolic status appears to be the most consistent determinant of human milk microbiota composition, whereas dietary and clinical factors exert more context-dependent effects. Across studies, metabolic dysregulation and inflammatory conditions are generally associated with reduced microbial diversity and altered abundance of key taxa such as *Bifidobacterium* and *Lactobacillus*, although variability remains across cohorts and study designs.

### 4.2 Delivery mode and lactation stage

The mode of delivery can influence human milk microbiota composition and diversity, particularly in early postpartum samples (Figure 2). Elective cesarean sections are often associated with decreased *Leuconostocaceae* and increased *Carnobacteriaceae*, whereas non-elective cesarean sections more closely resemble vaginal births (Cabrera-Rubio et al., 2012; Williams et al., 2017). Unscheduled cesarean deliveries may increase *Brevundimonas* abundance in milk collected at 3 months postpartum, whereas scheduled cesarean sections show lower levels compared with unscheduled procedures at the same time point (LeMay-Nedjelski et al., 2020). Overall, cesarean section may alter milk microbial composition, reduce alpha diversity and richness, and limit the presence of bacterial genera such as *Bifidobacterium*, which are primarily detected in vaginally delivered mothers in samples collected 1 month postpartum (Hermansson et al., 2019). Some populations also show higher Proteobacteria abundance in milk collected 1 month postpartum (Kumar et al., 2016). Probiotic supplementation appears more effective in increasing *Lactobacillus* and *Bifidobacterium* in vaginally delivering mothers than in cesarean mothers, regardless of whether colostrum or mature milk was analyzed (Mastromarino et al., 2015).

Early milk shows the most pronounced differences in total bacterial load and specific taxa such as *Streptococcus* and *Bifidobacterium*, which tend to diminish in mature milk (Khodayar-Pardo et al., 2014). Vaginally delivered colostrum generally shows higher richness of *Streptococcus* and *Haemophilus*, and lower abundance of *Pseudomonas*, *Staphylococcus*, *Prevotella*, *Finegoldia*, and *Halomonas*, reflecting differences in microbial networks between delivery modes (Toscano et al., 2017). However, some studies report minimal differences in dominant milk taxa between mothers with different delivery modes, particularly when milk was collected later postpartum (Urbaniak et al., 2016). For example, Urbaniak et al. analyzed milk collected from day 6 postpartum onwards, including both transitional and mature milk, and found that the relative abundances of most taxa did not change significantly over time, suggesting that the milk microbiota is relatively stable and resilient to delivery-associated hormonal or inflammatory variation.

The milk microbiota also evolves throughout lactation. Colostrum is typically dominated by *Weisella*, *Leuconostoc*, *Staphylococcus*, and *Streptococcus*. By 1 and 6 months postpartum, oral cavity-associated bacteria such as *Veillonella*, *Leptotrichia*, and *Prevotella* increase, while overall diversity decreases (Cabrera-Rubio et al., 2012). In some cohorts, minor increases in *Veillonella* and *Granulicatella* are observed between 4 and 6 months, although the overall microbiome remains relatively stable (Williams et al., 2017). Longitudinal analyses show a gradual decline in microbial diversity and richness from colostrum to transitional to mature milk, with increasing *Proteobacteria* and decreasing *Firmicutes* over time. *Staphylococcus* abundance declines, whereas *Streptococcus* remains relatively stable (Wan et al., 2020). Comparisons between early (10 days) and later (3 months) postpartum samples indicate reduced *Staphylococcus* and increased species richness, including *Rothia*, *Veillonella*, *Granulicatella*, and *Methylobacterium*, reflecting dynamic microbial evolution influenced by maternal physiology and infant feeding (Simpson et al., 2018). Other studies report progressive increases in total bacterial load and specific taxa including *Bifidobacterium*, *Enterococcus*, and *Lactobacillus*, suggesting microbial adjustment to infant developmental needs (Khodayar-Pardo et al., 2014). Longer lactation correlates with greater α-diversity and interindividual variation, marked by rising *Actinobacteria* and *Bacteroidetes* while *Firmicutes* remain stable (Sanjulián et al., 2021).

Collectively, these findings suggest that delivery mode primarily shapes initial mammary microbial seeding, with effects strongest in early milk. The observed temporal dynamics underscore a critical window for interventions, such as early probiotic supplementation, enhanced skin-to-skin contact, and breastfeeding support, particularly for cesarean-delivered mothers.

### 4.3 Infant-related and geographic/environmental factors

Infant characteristics exert subtle but measurable effects on milk microbiota (Figure 2). Infant sex has been reported in some studies to be associated with shifts in dominant bacterial taxa, rather than showing a consistent effect across the literature. Milk for male infants tends to contain higher *Streptococcus* and lower *Staphylococcus*, whereas the opposite pattern is observed in milk for female infants (Williams et al., 2017). Importantly, these findings derive from observational analyses and do not imply causality. These differences may result from bidirectional microbial exchange during breastfeeding, including retrograde flow of oral microbes and infant-derived hormonal or immune signals, suggesting a dynamic maternal-infant microbial dialogue in which the infant actively influences milk composition. However, other studies have not observed significant sex-related differences in milk microbiota composition, indicating that the influence of infant sex is likely modest, context-dependent, or sensitive to study design and analytical methods.

Gestational age also contributes to microbial variation. Milk from term pregnancies generally shows higher *Bifidobacterium* across lactation and lower *Enterococcus* in colostrum compared with preterm births, likely reflecting greater maternal gut maturity and more effective immune-mediated bacterial trafficking along the entero-mammary pathway (Khodayar-Pardo et al., 2014). In contrast, some studies report minimal differences in milk microbiota between term and preterm deliveries (Urbaniak et al., 2016), and broader comparative analyses across mammalian species indicate that certain milk microbial taxa and community structures are influenced by host evolutionary history and ecological factors (e.g., conserved patterns observed across diverse mammals) (Ge et al., 2021; Keady et al., n.d.). These findings suggest that specific milk microbial elements may be functionally conserved across mammals, supporting the transmission of beneficial bacteria and safeguarding neonatal colonization even under suboptimal perinatal conditions.

Geographic and environmental context further shapes milk microbiota. Ethnicity and region are consistently associated with compositional differences. *Corynebacterium* and *Brevundimonas* are less abundant in White mothers, whereas Asian mothers show lower *Corynebacterium* and *Aeromonas* (LeMay-Nedjelski et al., 2020). Cross-country comparisons reveal distinct microbial signatures that persist from colostrum to mature milk, including higher *Actinobacteria* in Chinese mothers and greater *Bacteroidetes* in Spanish cohorts (Kumar et al., 2016; Williams et al., 2017; Wan et al., 2020). These patterns likely reflect complex, interacting influences of diet, lifestyle, environmental microbial reservoirs, and sociocultural practices rather than direct causal effects of any single factor.

Collectively, infant characteristics and environmental context, alongside maternal factors, shape milk microbiota. While associations with infant sex and gestational age have been reported, current evidence suggests that these effects are generally modest and not consistently observed across studies, whereas geographic and environmental influences appear more robust. Geographic variation highlights the importance of contextualizing milk microbiota research within cultural and ecological frameworks rather than assuming a universal “healthy” milk microbiome. Future studies should integrate multi-omic and longitudinal approaches to disentangle how infant-driven feedback, maternal physiology, and environmental influences interact to shape milk microbial ecology and downstream infant health outcomes, including immune maturation and disease susceptibility.

### 4.4 Milk components

Human milk composition is a major factor shaping milk microbiota, creating a dynamic environment that selectively favors or inhibits specific bacterial taxa (Figure 2). Lipids play a particularly important role. Monounsaturated fatty acids are negatively associated with *Proteobacteria* and *Lactobacillus*, whereas PUFAs generally support beneficial microbes (Kumar et al., 2016). These observations suggest that variations in fatty acid profiles may generate microenvironments that either constrain or promote the growth of particular microbial groups, potentially influencing the functional properties of milk microbiota.

Polyamines, including putrescine, spermidine, and spermine, also modulate microbial growth. For example, putrescine correlates positively with *Gammaproteobacteria* and *Pseudomonas fragi* (Gómez-Gallego et al., 2017), demonstrating that even minor bioactive molecules can shape selective niches within milk.

HMOs are among the best-characterized modulators of milk microbiota. They act as selective carbon sources, promoting the growth of *Bifidobacterium* and influencing bifidobacterial communities in the infant gut through cross-feeding of metabolic by-products such as fucose, acetate, pyruvate, and 1,2-propanediol (Lawson et al., 2020). Specific oligosaccharide types are associated with particular bacterial taxa. Higher total concentrations correlate with increased *Bifidobacterium*, sialylated oligosaccharides with *Bifidobacterium breve*, non-fucosylated and non-sialylated oligosaccharides with the *Bifidobacterium longum* group, fucosylated oligosaccharides with *Akkermansia muciniphila*, and fucosylated-sialylated oligosaccharides with *Staphylococcus aureus* (Aakko et al., 2017). These patterns indicate that oligosaccharide diversity not only determines which microbes thrive in milk but may also indirectly shape infant gut colonization and early immune development.

Minerals and fatty acids further contribute to shaping milk microbial communities. *Streptococcus* and *Staphylococcus* abundances are associated with calcium, magnesium, and selenium, whereas *Proteobacteria* positively correlate with docosahexaenoic acid. *Staphylococcus* shows a positive association with conjugated linoleic acid, whereas *Streptococcus* is negatively associated with trans-palmitoleic acid (Sanjulián et al., 2021).

Overall, milk functions as a complex ecological system in which nutrients and bioactive compounds drive microbial assembly, implying that maternal diet, physiology, and metabolism can have cascading effects on milk microbiota and infant microbial colonization.

### 4.5 Probiotic supplementation and interventions

Maternal probiotic supplementation represents a promising strategy to modulate human milk microbiota (Figure 2), although its effectiveness depends on the specific probiotic strains used, maternal context, and delivery mode. Supplementation with *Lactobacillus rhamnosus* GG, *Lactobacillus acidophilus* La-5, and *Bifidobacterium animalis* subsp. *lactis* Bb-12 did not produce significant changes in overall milk microbial composition, suggesting limited capacity of these strains to colonize the mammary gland via entero-mammary transfer (Simpson et al., 2018).

In contrast, administration of the multistrain probiotic VSL#3 during late pregnancy and lactation increased *Lactobacillus* and *Bifidobacterium* levels in both colostrum and mature milk of vaginally delivered mothers, while showing no detectable effect in cesarean-delivered mothers (Mastromarino et al., 2015). This finding indicates that delivery mode may modulate the ability of probiotics to influence milk microbiota.

Maternal intake of *Lactobacillus rhamnosus* or *Saccharomyces boulardii* over a 2-month period enhanced *Lactobacillus* and *Bifidobacterium* abundance in milk and shifted the relative proportions of other genera, including *Streptococcus*, *Staphylococcus*, *Veillonella*, and *Bacteroides* (Shin et al., 2021). These interventions increased species diversity and were associated with dynamic changes in the infant fecal microbiota. To date, only one RCT has directly examined the effects of prebiotics on the human milk microbiota. Maternal supplementation with fructo-oligosaccharides (FOS) during lactation did not substantially alter the overall structure of the milk microbiota (Padilha et al., 2020). However, these findings are preliminary, and further well-designed studies are needed to confirm the impact of prebiotics on milk microbial composition.

Collectively, these results highlight that probiotic interventions can shape milk microbial communities, with effectiveness influenced by strain characteristics, maternal factors, and delivery mode. These findings support the potential for targeted maternal probiotic strategies to modulate milk microbiota and, potentially, infant gut colonization.

### 4.6 Maternal antibiotic exposure

Maternal antibiotic use can significantly alter human milk microbiota by disrupting maternal gut and mammary microbial communities and impairing the entero-mammary transfer of beneficial bacteria (Figure 2). Such disruptions may have downstream consequences for infant gut colonization and immune development, emphasizing the importance of judicious antibiotic use during pregnancy and lactation.

Administration of intrapartum antibiotics has been shown to modify milk microbiota composition, occasionally increasing diversity and richness, while often depleting key taxa such as *Bifidobacterium* (Hermansson et al., 2019). This represents a short-term, largely reversible perturbation, although selective reduction of critical beneficial taxa may affect the initial microbial seeding in infants. Antibiotic exposure during pregnancy or lactation consistently reduces the presence of *Lactobacillus* and *Bifidobacterium*, independent of delivery mode or lactation stage. Moreover, prophylactic antibiotics given around the time of labor transiently suppress *Bifidobacterium* during the first postpartum week, with levels generally recovering by 1 month (Padilha et al., 2019b). These findings emphasize the dual effect of maternal antibiotics: temporary disruptions that recover over time, alongside selective depletion of key beneficial bacteria. Interventions such as targeted probiotic supplementation may help mitigate these unintended impacts on milk microbiota.

### 4.7 Infant feeding mode

Infant feeding mode is a major determinant of human milk microbiome composition (Figure 2). Direct breastfeeding facilitates retrograde transfer of infant oral microbes into the mammary gland, enabling bidirectional microbial exchange between mother and infant (Laursen et al., 2021; Wu et al., 2025). This process promotes enrichment of infant oral-associated taxa, including *Streptococcus*, *Veillonella*, and *Haemophilus*, supporting microbial diversity and functional stability. In contrast, pumped or bottle-fed milk shows reduced exposure to infant oral microbiota and greater influence from environmental sources, which may be associated with lower abundances of beneficial taxa such as *Bifidobacterium* and *Lactobacillus* (Notarbartolo et al., 2022). Milk expressed via a pump has been reported to contain lower levels of cultivable staphylococci but higher total bacterial DNA compared with manually expressed milk (Treven et al., 2019). Moreover, pumped milk is characterized by reduced *Bifidobacterium* and increased *Enterobacteriaceae* and *Pseudomonadaceae*, whereas direct breastfeeding is associated with enrichment of *Gemellaceae*, *Vogesella*, and *Nocardioides* (Moossavi et al., 2019). Collectively, these findings highlight feeding mode and milk expression method as key determinants of milk microbiota composition with potential implications for early infant gut colonization.

## 5 Human milk microbiota: implications for infant microbiome development

### 5.1 Establishment of the infant oral microbiota

**FIGURE 3:**
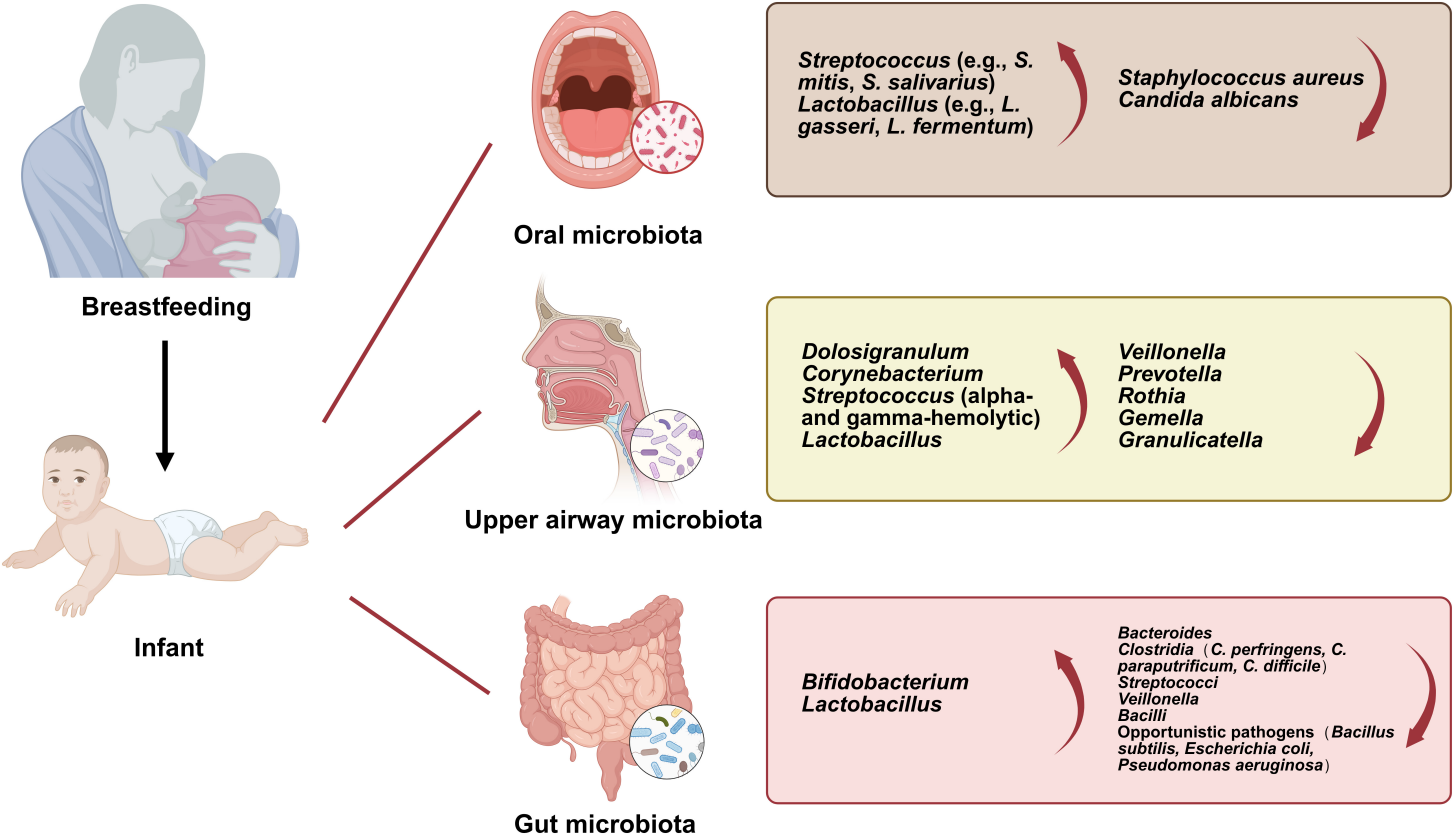
Impact of breastfeeding on infant oral, airway, and gut microbiota. Breastfeeding is associated with more stable and diverse microbiota. In the oral cavity, dominant genera include *Streptococcus* (e.g., *S. mitis*, *S. salivarius*) and *Lactobacillus* (e.g., *L. gasseri*, *L. fermentum*), with lower levels of potential pathogens such as *Staphylococcus aureus* and *Candida albicans*. In the upper airway, dominant genera include *Dolosigranulum*, *Corynebacterium*, and *Streptococcus* (alpha- and gamma-hemolytic), with higher levels of lactic acid bacteria and lower abundance of opportunistic anaerobes and potential pathogens (*Veillonella*, *Prevotella*, *Rothia*, *Gemella*, *Granulicatella*) compared with formula-fed infants. In the gut, breastfed infants are dominated by *Bifidobacterium* (particularly *B. breve*) and *Lactobacillus*, exhibit higher microbial diversity, and have lower abundances of opportunistic pathogens such as *Clostridium perfringens*, *C. paraputrificum*, *C. difficile*, *Bacillus subtilis*, *Escherichia coli*, and *Pseudomonas aeruginosa*, whereas formula-fed infants show increased counts of *Bacteroides*, *Clostridia*, *Enterobacteria*, *Streptococci*, *Veillonella*, and *Bacilli*.

Breastfeeding has been consistently associated with more stable and diverse infant oral, respiratory, and gut microbiota (Figure 3). The infant oral microbiota is initially seeded by maternal microbes, particularly from precolostrum and early breast milk, which provide live bacteria, nutrients, and bioactive compounds that support early colonization and long-term oral health (Martín et al., 2007; Kaan et al., 2021). Precolostrum, collected before infant contact, harbors diverse bacteria from the maternal oral cavity (*Streptococcus*, *Veillonella*, *Rothia*), skin (*Staphylococcus*, *Corynebacterium*), and gut (*Bifidobacterium*, *Prevotella*) (Ruiz et al., 2019a). Whole-genome sequencing of mother–infant pairs demonstrates that strains in precolostrum often match those in the infant oral cavity with >99.9% nucleotide identity, supporting direct maternal–infant microbial transmission (Ruiz et al., 2019a).

During breastfeeding, maternal microbes are continuously delivered via milk and repeated nipple-oral contact. *Streptococcus* and *Staphylococcus* consistently establish early dominance, whereas milk-derived *Bifidobacterium* transits the oral cavity without long-term colonization (Biagi et al., 2017). These pioneer species contribute to the establishment of a stable oral community and may influence subsequent gut microbiota development. Feeding method modulates oral bacterial composition, as *Lactobacillus* species are more frequently detected in breastfed than formula-fed infants, with 27.8% of exclusively or partially breastfed infants harboring *Lactobacillus*, compared with none of the formula-fed infants (Holgerson et al., 2013). At the phylum level, breastfeeding is associated with higher abundances of *Actinobacteria* and *Proteobacteria*, while formula feeding favors *Bacteroidetes* (Al-Shehri et al., 2016). At the genus level, breastfed infants are enriched in *Streptococcus*, whereas formula-fed infants show higher *Actinomyces* and *Prevotella* abundance. Overall species richness increases with age, while fungal communities remain relatively stable, highlighting the combined effects of diet and maturation on oral microbiota development (Oba et al., 2020).

In the first 2 months of life, *Streptococcus*, *Lactobacillus*, and *Staphylococcus* dominate the oral microbiota, reflecting taxa commonly found in human milk (García-Quintana et al., 2023). Although differences by delivery mode and feeding practice are generally modest, exclusively breastfed infants exhibit more stable and diverse oral communities. Breast milk thus serves as a continuous source of maternal microbes, facilitating oral microbial colonization and potentially compensating for early life microbial deficits, particularly in cesarean-delivered infants.

Breastfeeding also promotes colonization by beneficial *Lactobacillus* species, notably *Lactobacillus gasseri*, detected in 34% of breastfed but < 10% of formula-fed infants (Romani Vestman et al., 2013). *Lactobacillus gasseri* adheres to oral epithelial cells and salivary components (gp340, MUC7, MFGM) and inhibits key oral pathogens such as *Streptococcus mutans* and *Candida albicans*. These findings indicate that breast milk not only supports early oral microbial assembly but also selectively enhances colonization by protective species, contributing to microbiota stability and defense against pathogens.

Collectively, human milk acts as a continuous reservoir of maternal microbes, particularly *Lactobacillus* and *Streptococcus*, supporting early oral microbial colonization, enhancing microbiota stability and diversity, and potentially shaping subsequent gut microbiota development.

### 5.2 Establishment of the infant gut microbiota

The infant gut microbiota is established through a dynamic process beginning at birth and is strongly influenced by early life exposures, with human milk microbiota playing a pivotal role. Maternal microbes are transmitted via multiple pathways, including vaginal and gut microbiota exposure during birth, direct breastfeeding, and contact with maternal skin and the surrounding environment (Penders et al., 2006; Ma et al., 2023). Estimates suggest that approximately 27% of the infant gut microbiota is derived from maternal milk bacteria (Wang et al., 2021).

Breast milk is enriched in genera such as *Acinetobacter*, *Stenotrophomonas*, *Sphingopyxis*, *Pseudomonas*, and *Streptococcus*, whereas infant feces are dominated by *Bifidobacterium*, *Escherichia-Shigella*, *Klebsiella*, *Streptococcus*, *Serratia*, *Bacteroides*, and *Lactobacillus*. Certain taxa, including *Acinetobacter*, *Bifidobacterium*, *Klebsiella*, and *Lactobacillus*, are shared between milk and infant feces, indicating a potential maternal origin (Wang et al., 2023). Overall, milk microbiota, dominated by *Firmicutes* and *Actinobacteriota*, correlates with infant gut composition, particularly in exclusively breastfed infants. Key taxa such as *Lactobacillus* and *Bifidobacterium* likely influence the gut indirectly via SCFAs -mediated cross-feeding rather than direct strain colonization (Li Y. et al., 2022).

Infants consuming bacterial-containing breast milk exhibit higher gut microbial diversity and increased abundances of *Lachnospirales*, *Lachnospiraceae*, and *Eggerthellaceae*, along with more complex microbial networks, whereas maternal clinical characteristics and milk composition do not differ between sterile and bacterial-containing milk (Huang et al., 2022). Feeding type strongly shapes early gut microbiota: in breastfed infants, *Bifidobacterium*, particularly *B. breve*, rapidly dominates, whereas formula-fed infants show lower *Bifidobacterium* levels and higher abundances of *Bacteroides*, *Clostridia*, *Enterobacteria*, *Streptococci*, *Veillonella*, *Bacilli*, and opportunistic pathogens such as *Clostridium perfringens*, *C. paraputrificum*, *C. difficile*, *Bacillus subtilis*, *Escherichia coli*, and *Pseudomonas aeruginosa* (Yoshioka et al., 1983; Benno et al., 1984). These findings indicate that breast milk not only promotes colonization by beneficial microbes but also constrains the establishment of potentially pathogenic taxa, supporting healthy infant gut development.

### 5.3 Establishment of the infant airway microbiota

The establishment of the infant airway microbiota is a pivotal process in early life, shaping airway immunity and susceptibility to infections. From birth, the upper airway is rapidly colonized by a dynamic bacterial community that undergoes temporal succession, influenced by host, environmental, and maternal factors (Biesbroek et al., 2014b; Bosch et al., 2017; Thorsen et al., 2019; de Steenhuijsen Piters et al., 2020; Pirr et al., 2025). While initial colonization may begin prenatally, it primarily occurs postnatally, with the nasopharynx and oropharynx as the main sites. Pioneer bacteria such as *Staphylococcus* appear in the hypopharynx within the first weeks of life (Biesbroek et al., 2014b; Mortensen et al., 2016).

During infancy, the upper airway microbiota follows a defined succession. *Staphylococcus* is initially dominant but is gradually replaced by genera including *Moraxella*, *Streptococcus*, and *Haemophilus*. This succession is closely associated with infection risk. In healthy infants, the nasopharyngeal microbiota reaches relative stability by approximately 12 months. Feeding mode further modulates this process, influencing pathogen prevalence and long-term respiratory outcomes (Biesbroek et al., 2014b; Mortensen et al., 2016).

A study of healthy breastfed and formula-fed infants found that pathogenic bacteria were less frequently isolated from breastfed infants (Hokama et al., 1996). More recent sequencing-based research has confirmed that breastfed infants typically harbor lower abundances of several opportunistic/pathogenic taxa compared with formula-fed infants (Ma et al., 2020). In both groups, alpha- and gamma-hemolytic *Streptococcus* dominated the throat microbiota. Early cessation of breastfeeding (before 3 months) accelerates premature acquisition of microbial species and metabolic pathways. For instance, the premature acquisition of *Ruminococcus gnavus* and early life alterations in tryptophan metabolism have been associated with immune modulation and an increased risk of asthma development (Shenhav et al., 2024). Conversely, exclusive breastfeeding for over 3 months supports gradual microbiome maturation and protects against preschool asthma. Colonization patterns of nasal and gut microbiota, together with human milk composition, predict asthma risk.

By 6 weeks of age, breastfed infants exhibit a nasopharyngeal microbiota distinct from that of formula-fed infants. One study reported higher abundances of *Dolosigranulum* and *Corynebacterium* and lower abundances of *Staphylococcus*, *Prevotella*, and *Veillonella* in breastfed infants (Biesbroek et al., 2014a). Another study observed broader differences. Breastfeeding was associated with higher levels of lactic acid bacteria and lower levels of *Staphylococcus* and anaerobic genera. Formula-fed infants exhibited greater abundances of anaerobes including *Veillonella* and *Prevotella*, as well as genera such as *Streptococcus*, *Rothia*, *Gemella*, and *Granulicatella* (Biesbroek et al., 2014b).

Although the mechanisms remain incompletely understood, milk-derived lactic acid bacteria, including *Lactobacillus gasseri* and *Streptococcus* strains, may inhibit pathogen growth, adhere to epithelial surfaces, and modulate local immunity. Other milk-associated taxa, such as *Veillonella*, *Rothia*, and *Corynebacterium*, may also contribute to early upper airway microbiota assembly and functional resilience. Collectively, these data underscore that human milk provides both well-studied protective strains and a broader microbial community, promoting airway microbiota maturation and potentially influencing long-term respiratory health.

## 6 Human milk-mediated transmission of maternal microbiota to infants

**FIGURE 4:**
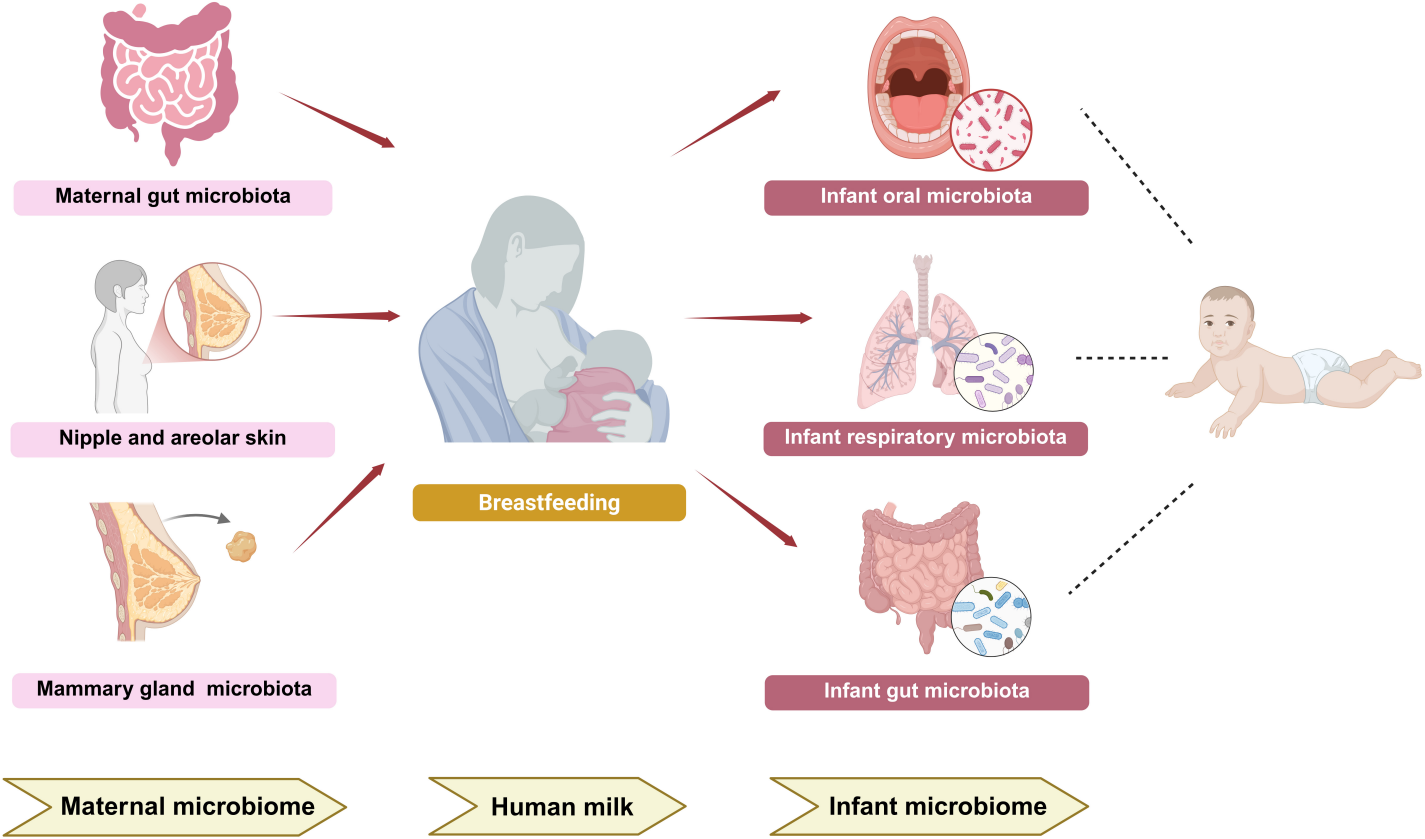
Maternal microbial transfer via human milk. Human milk contains diverse commensal and beneficial bacteria that support early infant microbiome development. Maternal microbes are delivered through multiple routes, including nipple and areolar skin during breastfeeding, translocation from the maternal gut via the entero-mammary pathway, and resident mammary duct microbiota. These microbes colonize the infant’s oral, respiratory, and gut sites, contributing to microbiome establishment, immune maturation, and long-term health.

Human milk-mediated transmission of maternal microbiota represents a proposed mechanism by which commensal and beneficial bacteria from the mother may contribute to shaping the infant microbiome (Figure 4). Human milk is not merely a nutrient source; it also delivers a diverse array of commensal microbes that are thought to play a role in early microbial colonization. Culture-independent and strain-resolved analyses have reported that taxa such as *Bifidobacterium*, *Lactobacillus*, and *Staphylococcus* are shared between milk and the infant gut, and may contribute to colonization during the first month of life (Duranti et al., 2017; Pannaraj et al., 2017; Lundgren et al., 2023).

Maternal microbes may reach milk and the infant gut via multiple, non-exclusive routes. These include transfer from maternal skin and areolar sites during breastfeeding, potential translocation from the maternal gut via a putative entero-mammary pathway, in which live microbes or microbial DNA may be transported by immune cells, and contributions from resident mammary duct microbiota during late pregnancy and lactation (Geddes et al., 2008; Jiménez et al., 2008; Latuga et al., 2014; de Andrés et al., 2017; Duranti et al., 2017). Current evidence indicates that skin- and oral-derived taxa are most consistently detected, and accumulating molecular and cellular findings now support a putative entero-mammary pathway through which microbial cells or microbial DNA may be trafficked from the maternal gut to the mammary gland. Transfer via these routes may deliver both live bacteria and microbial metabolites, potentially contributing to early immune priming of the infant before gut colonization is fully established.

Several lines of evidence suggest a role of human milk in shaping the infant microbiota. The abundance of specific maternal microbes in milk correlates with higher colonization in the infant gut, suggesting a dose-dependent effect (Shama et al., 2024). HMOs and other nutrients selectively promote the growth of these beneficial bacteria while limiting pathogens, providing both microbial and metabolic support for early colonization (Laursen et al., 2021; Zhang S. et al., 2021). Maternal milk can partially restore microbiome composition in infants delivered by cesarean section (Liu et al., 2019; Guo et al., 2020). Moreover, higher maternal milk microbial diversity is associated with improved infant immune maturation and reduced risk of allergic or inflammatory conditions, indicating functional relevance beyond mere colonization (Fang et al., 2024).

Collectively, these findings support a model in which human milk may function as both a nutritional and microbial conduit, contributing to early infant microbiome development. This framework shifts maternal-infant microbial transfer from an observational correlation to a mechanistic and functional perspective, although direct causal evidence remains limited. Key questions remain regarding the long-term persistence of milk-derived strains, the precise mechanisms of the entero-mammary pathway, and the influence of maternal interventions such as diet or probiotics. Future longitudinal and interventional studies may help clarify whether targeted maternal probiotic supplementation, modulation of oligosaccharide composition, or controlled exposure to skin-derived commensals may optimize infant microbiota, immune maturation, and long-term health outcomes.

## 7 Clinical and translational perspectives: harnessing human milk microbiota to address postnatal microbiome colonization deficiencies

Early life microbial colonization is strongly influenced by delivery mode, gestational age, and perinatal antibiotic exposure. These factors can disrupt maternal-infant microbial transfer and compromise the establishment of a healthy microbiome. Human milk microbiota and its diverse bioactive components provide a biologically tailored means of restoring microbial balance in these contexts. This section highlights key clinical scenarios including cesarean birth, prematurity, and antibiotic exposure, and discusses how breastfeeding, together with targeted probiotic, prebiotic, and diet-based maternal interventions, can mitigate postnatal microbiome colonization deficiencies and support healthier early life trajectories.

### 7.1 Cesarean delivery: disrupted maternal microbial transmission and milk-mediated compensation

Cesarean-delivered infants often exhibit altered oral and gut microbiota, characterized by reduced diversity and delayed colonization by key commensal species (Dominguez-Bello et al., 2010; Rutayisire et al., 2016; Mueller et al., 2017, 2021; Fouhy et al., 2019; Shao et al., 2019). This microbial immaturity has been associated with increased susceptibility to infections, immune dysregulation, and metabolic disturbances (Dominguez-Bello et al., 2010; Kim et al., 2020; Zhang C. et al., 2021). Vaginally delivered infants typically harbor higher abundances of oral taxa such as *Lactobacillus*, *Prevotella*, and *Gardnerella*, whereas cesarean-born infants show elevated levels of *Petrimonas*, *Bacteroides*, *Desulfovibrio*, *Pseudomonas*, *Staphylococcus*, and *Bifidobacterium* (Lif Holgerson et al., 2011; Inchingolo et al., 2024). Similarly, cesarean-born infants exhibit persistently lower gut microbial diversity, delayed colonization by beneficial taxa (Jakobsson et al., 2014; Rutayisire et al., 2016; Coker et al., 2021; Ma et al., 2023), impaired vertical transmission of maternal *Bacteroides*, and increased prevalence of opportunistic hospital-associated pathogens, including *Enterococcus*, *Enterobacter*, and *Klebsiella* (Jakobsson et al., 2014; Shao et al., 2019; Inchingolo et al., 2024).

Cesarean-born infants bypass the birth canal, losing direct exposure to maternal vaginal and gut microbiota, which are critical for early gut colonization. Under normal conditions, vaginal delivery, together with maternal gut, provides the primary microbial inoculum for the neonate (Ferretti et al., 2018; Bogaert et al., 2023). To mitigate the disrupted microbial transmission, “vaginal seeding” has been proposed, wherein sterile swabs soaked with maternal vaginal secretions are applied to the infant’s lips, face, and body. Some studies indicate that this approach can partially align the gut microbiota composition of cesarean-born infants with that of vaginally delivered infants (Dominguez-Bello et al., 2016; Song et al., 2021). However, the American College of Obstetricians and Gynecologists (ACOG) does not recommend vaginal seeding as routine clinical practice due to potential pathogen transmission risks (e.g., group B *Streptococcus*, herpes simplex virus, *Neisseria gonorrhoeae*) (Vaginal Seeding, n.d.). The American Academy of Pediatrics (AAP) also maintains a cautious stance, suggesting that it should only be conducted in strictly controlled research settings with thorough maternal infection screening to minimize risk (Nolt et al., 2022). Moreover, attempts to orally administer maternal vaginal microbiota, as an alternative to vaginal seeding, have not demonstrated significant effects on early gut microbial development (Wilson et al., 2021). Moreover, multiple studies emphasize that maternal gut microbiota is the dominant source for neonatal gut colonization, whereas vaginal microbiota plays only a secondary and transient role (Sakwinska et al., 2017; Ferretti et al., 2018; Li et al., 2024; Meng et al., 2025; Ronde et al., 2025; Xie H. et al., 2025), further undermining the rationale for vaginal seeding as an effective intervention. Taken together, these findings indicate that vaginal seeding is not an ideal or recommended intervention.

Maternal fecal microbiota transplantation (FMT) may represent a more direct approach to supplement gut-derived microbes in cesarean-born infants (Korpela et al., 2020; Helve et al., 2021). However, its safety, feasibility, optimal dosage, and clinical applicability remain to be systematically evaluated. Future research is needed to determine whether FMT can serve as a reliable intervention for restoring early life gut microbial development in high-risk neonates.

**FIGURE 5:**
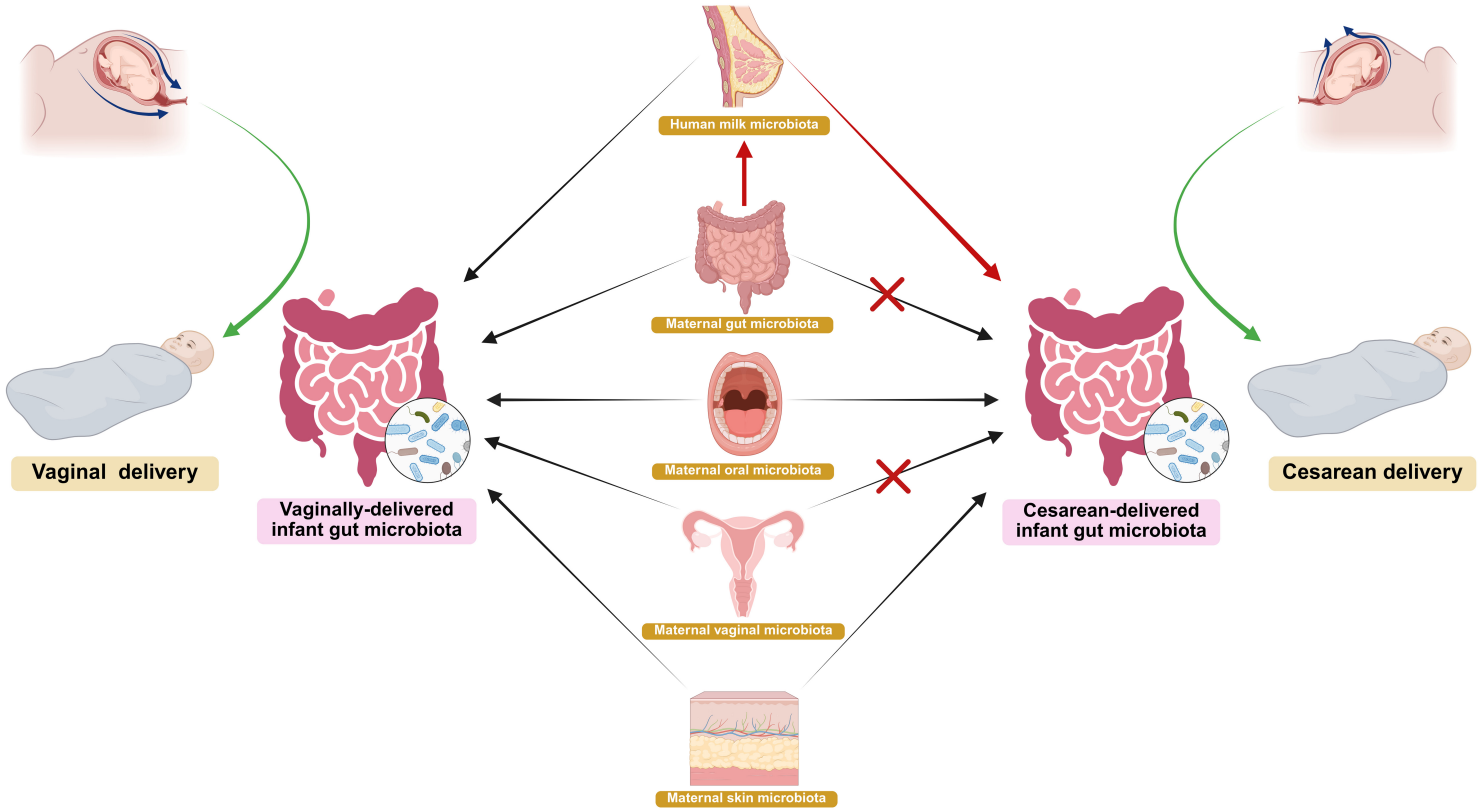
Maternal milk-mediated compensation for gut microbiota deficits in cesarean-delivered neonates. In vaginally delivered neonates, maternal microbes are transferred from multiple sources, including the maternal vaginal tract, gut, milk, oral cavity, and skin. In contrast, cesarean-delivered neonates exhibit inherent deficiencies in maternal vaginal and gut microbiota. Remarkably, these deficits may can be partially compensated postnatally through maternal milk, which delivers gut-derived microbes to the infant and helps restore microbial colonization.

Human milk functions as a key mediator for maternal microbiota transmission to infants, with a significant portion of its microbial content originating from the maternal gut. Through breastfeeding, maternal gut microbes and probiotics, including beneficial genera such as *Lactobacillus* and *Streptococcus*, are delivered directly to the infant, facilitating early gut colonization and the establishment of a healthy microbial ecosystem. This process is particularly critical for cesarean-born infants, who miss the microbial exposure associated with vaginal delivery; extended breastfeeding can partially restore their gut microbial profile toward that of vaginally delivered infants (Liu et al., 2019; Guo et al., 2020; Coker et al., 2021; Bogaert et al., 2023). The compensatory role of breast milk in cesarean-born infants is illustrated in Figure 5. These findings highlight breast milk as the primary natural and clinically safe route for cesarean-born infants to acquire maternal gut microbiota.

Beyond microbial delivery, breast milk provides various non-bacterial nutrients, including immune factors, lactose, oligosaccharides, lipids, and proteins, which can regulate gut ecology, promote the growth of beneficial bacteria, and support neonatal immune and metabolic development (Donovan and Comstock, 2016; van den Elsen and Verhasselt, 2021; Andres et al., 2023). Collectively, these insights underscore the irreplaceable role of breastfeeding in maintaining microbial diversity and overall health in cesarean-born infants and suggest that human milk represents a practical foundation for designing microbiota-targeted interventions in early life.

### 7.2 Preterm infants: compounded microbiome colonization deficiencies and milk-mediated restoration

Gestational age strongly influences intestinal microbiota, particularly in preterm infants. Earlier gestational age is associated with lower microbial diversity and delayed colonization by beneficial taxa, such as *Bifidobacteria* and *Bacteroides*, while opportunistic pathogens, including *Enterobacter*, *Enterococcus*, *Corynebacterium*, *Klebsiella*, and *Clostridium*, are more abundant (Mai et al., 2013; Drell et al., 2014; Arboleya et al., 2015; Gregory et al., 2016; Chernikova et al., 2018; Xie Z. et al., 2025). Preterm infants also exhibit reduced levels of microbiota-derived metabolites, such as SCFAs, which may impair immune development and increase susceptibility to diseases like necrotizing enterocolitis (NEC) (Bergström et al., 2014; Aguilar-Lopez et al., 2021).

Prenatal and postnatal antibiotic exposure-particularly common among preterm infants in neonatal intensive care units-can further disrupt gut microbiota and exacerbate deficits associated with delivery mode (Zou et al., 2018). Preterm infants delivered by cesarean section are particularly high-risk, as they experience the compounded effects of prematurity, altered birth mode, and frequent antibiotic exposure, leading to pronounced microbial colonization defects.

Breastfeeding promotes higher gut microbial alpha diversity in preterm infants, enriching key taxa such as *Bifidobacterium*, *Clostridiales*, *Lactobacilli*, and *Bacilli* (Cong et al., 2016, 2017; Alcon-Giner et al., 2020; Shama et al., 2024; Quigley et al., n.d.). This has been associated with reduced risks of NEC and sepsis. Additionally, the transition from tube feeding to oral or breastfeeding helps shape the oral microbiome, as evidenced by an increase in *Streptococcus* and a decrease in *Staphylococcus* following the initiation of oral feeding or breastfeeding (Schulkers Escalante et al., 2025). Elevated *Streptococcus* abundance is considered a hallmark of oral microbial maturation (Hendricks-Muñoz et al., 2015). In addition to affecting infant gut microbiota, prematurity alters human milk composition and diversity. Preterm breast milk exhibits increased species richness and higher abundances of *Staphylococcus haemolyticus*, *Propionibacterium acnes*, and unclassified *Corynebacterium* species (Singh et al., 2023), potentially weakening its capacity to support microbial colonization in preterm infants.

Encouraging early oral and breastfeeding may thus stabilize microbial communities and limit colonization by pathogenic bacteria. In addition, for mothers, breastfeeding provides protection against breast cancer, improves birth spacing, and may reduce the risk of ovarian cancer and type 2 diabetes, benefiting both maternal and infant health (Chowdhury et al., 2015; Pinho-Gomes et al., 2021).

### 7.3 Antibiotic exposure: microbiota disruption and milk-mediated resilience

Antibiotic exposure during delivery reduces gut bacterial diversity, decreases *Actinomycetes* (notably *Bifidobacteriaceae*), and increases *Proteobacteria*, while early postnatal antibiotics in full-term infants lower *Lactobacillus* and *Bifidobacteria* levels and elevate *Enterobacteria* (Arboleya et al., 2015; Zhou et al., 2020; Zimmermann and Curtis, 2020; Morreale et al., 2023). Even under such antibiotic exposure, breastfeeding enriches *Bifidobacterium longum* subsp. *infantis* in the infant gut, providing protection against asthma and other immune-mediated conditions (Dai et al., 2023). In addition, mouse models have shown that perinatal antibiotic exposure can impair intestinal epithelial proliferation and structural function, but administration of *Lactobacillus rhamnosus* GG can partially restore intestinal structure and function (Cuna et al., 2023).

Notably, this restorative effect of breastfeeding may be especially beneficial for infants exposed to antibiotics after birth, although it may be partially attenuated if mothers received antibiotics during pregnancy and delivery. This is because antibiotics during pregnancy and delivery can disrupt maternal milk microbiota by reducing or temporarily suppressing beneficial bacteria such as *Bifidobacterium* and *Lactobacillus*, potentially limiting milk’s capacity to support the establishment and resilience of the infant gut microbiome (Soto et al., 2014; Hermansson et al., 2019; Padilha et al., 2019b). Moreover, maternal antibiotic use can both disrupt the milk microbiota and lead to antibiotic residues in breast milk (Nahum et al., 2006; Dinleyici et al., 2018; Brockway, 2024), which, when ingested by the infant, may exert a persistent impact on the gut microbiota. These findings underscore the profound influence of perinatal antibiotics on infant intestinal microbial development and the conditional role of breast milk in mediating microbial resilience.

### 7.4 Milk- and probiotic-mediated strategies for microbiota restoration

Numerous studies have demonstrated that probiotic supplementation promotes gut microbial restoration in preterm infants by increasing the relative abundance of beneficial taxa such as *Bifidobacterium* and *Lactobacillus*, while concurrently reducing the prevalence of potentially pathogenic species (Plummer et al., 2018, 2021; Alcon-Giner et al., 2020; Athalye-Jape et al., 2022; Chang et al., 2024; He et al., 2024; Vievermanns et al., 2024). Probiotic use has also been associated with lower mortality and morbidity among preterm and low-birth-weight infants (Morgan et al., 2020).

Cesarean-section delivery bypasses exposure to maternal vaginal and intestinal microbiota, leading to early gut colonization characterized by reduced microbial diversity, altered dominant taxa, and increased prevalence of potentially pathogenic species. Probiotic supplementation can partially mitigate these effects, bringing the microbiota composition closer to that observed in vaginally delivered infants (Yang et al., 2021; Carpay et al., 2022; Gong et al., 2023; Nieto et al., 2025). Additionally, probiotics support gut microbiota recovery following neonatal antibiotic exposure by reducing antibiotic resistance genes and multidrug-resistant pathogens, while promoting the restoration of typical early life microbial profiles (Kiu et al., 2025). Although antibiotics significantly reduce *Bifidobacterium* and *Lactobacillus* abundance, probiotics cannot fully restore microbial diversity. Nonetheless, concurrent probiotic supplementation with antibiotics is more effective than delayed supplementation, particularly in enhancing *Bifidobacterium* abundance (Zhong et al., 2021).

As previously discussed, breastfeeding remains a cornerstone strategy for optimizing infant microbiome development, particularly in high-risk contexts such as cesarean delivery, prematurity, maternal dysbiosis, or congenital microbial deficiencies. Evidence suggests that maternal probiotic intake during pregnancy enriches beneficial bacteria in breast milk (Shin et al., 2021; Zaidi et al., 2021; Alemu et al., 2023). Consequently, maternal-infant probiotic supplementation during breastfeeding may yield synergistic effects. Moreover, bioactive components in breast milk, such as HMOs, lactoferrin, and immunoglobulins, further enhance the functional efficacy of probiotics by supporting their growth and promoting gut colonization (Carr et al., 2021; Ma et al., 2022; Gallo et al., 2024; Sun et al., 2024).

**FIGURE 6:**
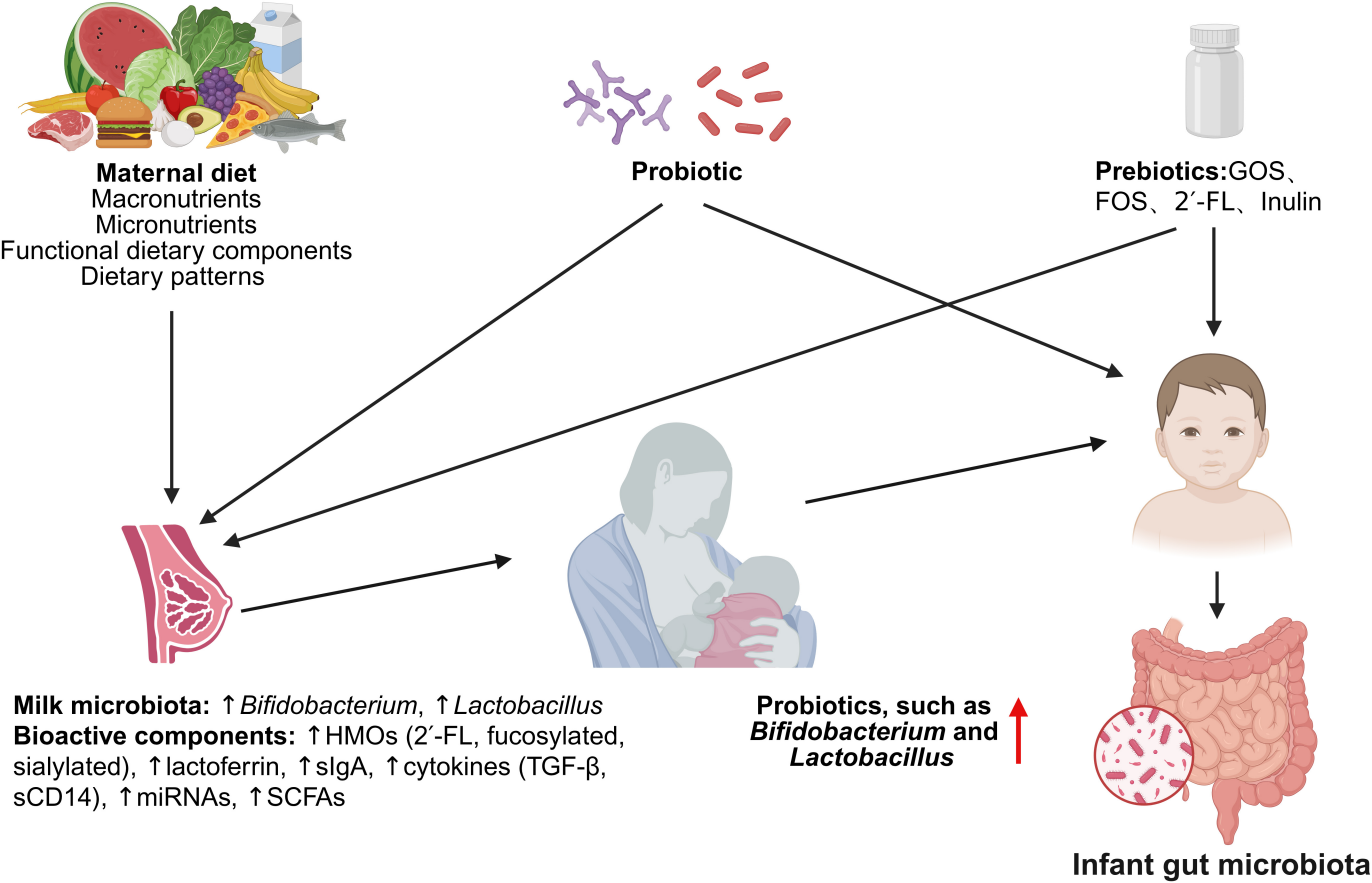
Infant microbiota restoration strategies mediated by maternal milk, probiotics, and prebiotics. Maternal interventions, such as probiotic supplementation, prebiotic intake, and dietary modulation, can shape milk microbiota composition and bioactive components (e.g., HMOs, lactoferrin, immunoglobulins, and microRNAs). Breastfeeding influences infant gut colonization, promoting beneficial taxa such as *Bifidobacterium* and *Lactobacillus* while reducing potential pathogens. Direct infant supplementation with probiotics or prebiotics may have synergistic effects in supporting healthy microbiota establishment and may help restore microbial balance in at-risk infants, including those born by cesarean section, preterm infants, and infants exposed to antibiotics.

Although some studies report minimal changes in the overall milk microbiome following maternal probiotic intake, accumulating evidence suggests that supplementation can induce significant modifications in immune and bioactive components. These include increases in secretory IgA, modulation of cytokines such as TGF-β, alterations in HMOs, and reductions in inflammatory markers (Baldassarre et al., 2016; Seppo et al., 2019; Takahashi et al., 2019, 2021; Zaidi et al., 2021; Taheri et al., 2022; Alemu et al., 2023; Gonia et al., 2024). Such changes may promote infant gut microbial development through mechanisms independent of major shifts in the milk bacterial community. Therefore, maternal probiotic supplementation during breastfeeding is still recommended, and infant probiotic administration may also be considered when appropriate. Together, these findings provide an integrated framework for understanding maternal-infant microbiota restoration strategies, as illustrated in Figure 6.

### 7.5 Milk- and prebiotic-mediated strategies for microbiota restoration

Prebiotics are substrates selectively utilized by beneficial gut microorganisms to improve host health, often described as “food for beneficial microbes” (Gibson et al., 2017; Davani-Davari et al., 2019; Deehan et al., 2024). Mechanistically, modulation of the maternal gut microbiota by prebiotics may indirectly shape the human milk microbiota, as the maternal gut serves as a key microbial reservoir for milk.

To date, only one randomized controlled trial has directly examined this relationship. Maternal supplementation with FOS during lactation did not substantially alter the overall structure of the milk microbiota (Padilha et al., 2020). Although no significant group-level differences were observed in major taxa, notable inter-individual variability was reported, and maternal age appeared to modulate responses to prebiotic intervention. Given the limited evidence, further research is needed to clarify the effects of prebiotics on the milk microbiota.

Beyond microbial composition, prebiotics may influence the milk microenvironment and maternal-infant health through immune factors (e.g., cytokines) and bioactive molecules such as microRNAs. For example, maternal supplementation with short-chain galacto-oligosaccharides (GOS) and long-chain FOS during pregnancy and lactation was associated with modulation of milk microbiota alongside altered concentrations of immune mediators, including TGF-β and sCD14, suggesting potential effects on infant immune development (Divakara et al., 2024). Animal studies further indicate that maternal FOS intake can regulate milk microRNA expression, correlating with offspring gut microbial composition and metabolic outcomes (Lowry et al., 2022).

Maternal prebiotic consumption also increases commensal *Bifidobacteria* in the maternal gut while reducing *Negativicutes* in both maternal and infant gut microbiota, accompanied by alterations in SCFA levels in maternal feces (Jones et al., 2024). Although these studies focused primarily on the gut, they demonstrate that prebiotic supplementation can influence maternal-infant microbial ecosystems and metabolic outputs. Consistent findings in animal models show that gestational supplementation with GOS or inulin increases *Bacteroidetes*, decreases *Firmicutes*, and enhances SCFA production (Brosseau et al., 2021). Given that SCFAs are detectable in human milk (Jiang et al., 2016; Prentice et al., 2019), maternal prebiotic intake may facilitate the transfer of gut-derived SCFAs into milk, potentially supporting infant gut health. While current evidence does not show consistent alterations in milk microbiota, the broader maternal and infant benefits associated with prebiotic-induced modulation of gut microbiota, immune mediators, and microbial metabolites suggest that maternal prebiotic supplementation is a biologically plausible and potentially beneficial strategy. High-quality clinical trials are needed to confirm these effects and define optimal intervention strategies.

Similarly, infant prebiotic supplementation promotes the establishment of beneficial gut bacteria. Early supplementation with GOS and FOS significantly increases key symbionts, particularly *Bifidobacterium*, and promotes a more mature metabolic profile resembling that of breastfed infants (Kapiki et al., 2007; Borewicz et al., 2019). Adding 2’-fucosyllactose (2’-FL, a HMO) to infant formula enhances the bifidogenic effect of prebiotics and demonstrates good safety and tolerance (Lazarini et al., 2025). Early prebiotic or synbiotic supplementation can partially correct the gut dysbiosis commonly observed in cesarean-delivered or preterm neonates by promoting colonization of beneficial taxa such as *Bifidobacterium* and *Lactobacillus* while suppressing potential pathogens (Lay et al., 2021; Carpay et al., 2022; Wang et al., 2025). This provides a safe and feasible early-life microbiota intervention for high-risk newborns. Maternal-infant co-supplementation with probiotics is also recommended.

Together, breastfeeding and prebiotic or probiotic supplementation may serve as complementary approaches to support microbial balance. These strategies have the potential to help alleviate, at least in part, the microbial disturbances associated with prematurity, altered birth mode, and early antibiotic exposure.

### 7.6 Milk- and diet-mediated strategies for microbiota restoration

Maternal diet is a modifiable factor that shapes milk microbiota composition and promotes early life colonization by beneficial bacteria. Macronutrients influence key microbial taxa: protein, PUFAs, and linoleic acid promote *Bifidobacterium*, whereas saturated and monounsaturated fatty acids (SFA, MUFA) and higher carbohydrate intake reduce taxa such as *Corynebacterium* and *Firmicutes*, suggesting selective modulation of the milk microbial community (Williams et al., 2017; Padilha et al., 2019a).

Micronutrients also affect milk microbes. Riboflavin and calcium intake enhance *Veillonella*, while vitamins D and E, chromium, and zinc reduce *Firmicutes*, *Lactobacillus*, and *Corynebacterium*, respectively, likely through effects on maternal metabolism and immune function (Williams et al., 2017; Padilha et al., 2019a). Vitamin C and fruit-derived compounds such as pectin and lycopene show similar trends, indicating that specific bioactive dietary compounds may influence microbial composition via gut–mammary interactions (Padilha et al., 2019a). Additionally, animal protein, total carbohydrates, vitamin A, and retinol are also linked to shifts in *Firmicutes* and *Bacteroidota*, reflecting nutrient-driven promotion of particular microbial groups (Bzikowska-Jura et al., 2025).

Beyond microbial composition, maternal diet can modulate milk bioactive components. A cross-over trial demonstrated that carbohydrate source (glucose vs. galactose) and energy source (high-fat vs. high-carbohydrate) significantly altered HMO profiles, particularly fucosylated and sialylated HMOs (Seferovic et al., 2020). These dietary effects may also influence microbial gene abundance, such as fucosidase, affecting functional microbial ecology. Dietary fiber, polyphenols, insoluble polysaccharides (e.g., pectin), and MUFAs positively correlate with HMO diversity, likely via modulation of glycosyltransferase activity in the mammary gland (Mokhtari et al., 2024). Higher maternal intake of vitamins D, C, K and minerals such as zinc and potassium is positively associated with 2’-FL and other HMO types (Li X. et al., 2022; Mokhtari et al., 2024). Conversely, high-fat diets reduced sialylated HMO concentrations (Biddulph et al., 2023).

Practical recommendations based on these findings suggest that maternal dietary interventions can be tailored according to metabolic profiles, taking into account factors such as genetics, BMI, and lifestyle, to optimize modulation of milk microbiota and HMO composition. Coordinated intake of macro- and micronutrients with bioactive compounds may enhance synergistic effects on milk microbial communities, promoting beneficial bacteria and functional milk components. Together, these strategies provide a practical approach to selectively enhance key microbial taxa, such as *Bifidobacterium* and *Lactobacillus*, and important milk bioactive components (e.g., HMOs), supporting early life microbial colonization and microbiome restoration.

### 7.7 Clinical translational strategies: leveraging human milk microbiota to restore postnatal microbial balance

Maternal breastfeeding remains the most accessible and clinically supported strategy for improving early life microbial establishment, particularly in high-risk neonates such as preterm infants, cesarean-born infants, or those exposed to perinatal antibiotics. Beyond providing a natural source of beneficial microbes and bioactive components, human milk represents a dynamic biological system that may be leveraged to support infant microbial colonization, immune signaling, and metabolic development.

Maternal-focused interventions represent an important but heterogeneous category of translational approaches with varying levels of evidence. Established evidence supports that breastfeeding and maternal nutritional optimization are associated with improved milk bioactive composition and infant microbial outcomes. Emerging evidence suggests that maternal probiotic supplementation during lactation may modulate milk microbial composition and potentially support infant gut colonization, although effects are strain-specific and not yet standardized. Evidence for prebiotic supplementation remains limited and indirect. Dietary optimization, including increased intake of prebiotic fibers, PUFAs, and selected micronutrients, may further influence milk microbiota and bioactive components such as HMOs, immunoglobulins, and antimicrobial peptides. Lifestyle-related factors, including metabolic regulation, physical activity, and prudent antibiotic use, may provide additional indirect modulation of milk composition. Collectively, these strategies are potentially feasible within routine prenatal and postnatal care, although their clinical efficacy requires further validation in well-controlled studies.

Milk- and infant-directed approaches provide complementary translational opportunities but remain largely in the emerging or experimental stage. Probiotic-enriched milk formulations and donor milk fortification offer standardized delivery systems for beneficial microbes, particularly in neonatal intensive care units (NICUs), where pasteurization may reduce microbial viability. However, regulatory approval, strain selection standardization, and safety evaluation, especially in preterm or medically fragile infants, remain important limitations. Similarly, incorporation of milk-derived probiotic strains from healthy lactating donors may offer biologically compatible candidates, but requires further evidence on safety, dosing, and colonization efficiency. Targeted supplementation with HMOs may selectively enrich *Bifidobacterium* species, although optimal formulations and clinical indications are still under investigation.

Technological advances further expand these possibilities. Microbiota-preserving processing methods, microbiota-enriched donor milk, and bioactivity-preserving fortifiers are being explored to mitigate microbial and functional loss during milk processing. Precision approaches matching specific probiotic strains or HMOs to infant clinical profiles represent a promising but still hypothetical frontier in personalized microbiome-based nutrition. Nonetheless, feasibility, cost-effectiveness, and equitable access across different healthcare systems remain important considerations for future implementation.

Overall, human milk functions both as a natural conduit for microbial transfer and as a potential platform for microbiome-informed interventions. While breastfeeding promotion remains foundational and evidence-based, most adjunct strategies-including maternal probiotic supplementation, microbiota-enriched formulations, and precision fortification-are still emerging and require further clinical validation. Regulatory, safety, and implementation challenges must be addressed before widespread clinical translation can be achieved.

## 8 Challenges and future directions

Despite substantial progress, the mechanisms linking maternal milk microbiota to infant health remain incompletely defined, in part due to persistent methodological challenges inherent to low-biomass microbiome research. A key limitation is the risk of contamination from reagents, the environment, and sample handling, which may obscure true milk-associated microbial signals. In addition, DNA-based sequencing approaches cannot reliably distinguish viable, metabolically active bacteria from non-viable microbial DNA, limiting causal interpretation of functional colonization.

Future research will benefit from longitudinal multi-omic designs integrating maternal gut, milk, and infant microbiomes with metabolomic and immunologic data. These approaches should be combined with stricter contamination control and, where feasible, methods to assess microbial viability. Systematic evaluation of maternal interventions across preconception, pregnancy, and lactation is also required to clarify their ability to enrich beneficial taxa such as *Bifidobacterium* and *Lactobacillus*, particularly in high-risk neonates. Key unresolved issues include the existence and functional relevance of the entero-mammary pathway, the persistence of milk-derived strains in the infant gut, and their downstream effects on immune, metabolic, and neurodevelopmental outcomes.

Methodological variability further contributes to inconsistent findings across studies. Differences in sequencing depth, primer selection, DNA extraction protocols, and bioinformatic pipelines introduce analytical bias and limit cross-study comparability. Standardized sampling and analytical frameworks are therefore essential to distinguish true biological signals from methodological noise across diverse populations and environments.

Importantly, most available evidence derives from high-income countries, which may limit generalizability to low- and middle-income populations. Differences in maternal diet, environmental exposure, socioeconomic conditions, and breastfeeding practices may further influence milk microbiota and infant outcomes, highlighting the need for more geographically and culturally diverse studies.

Translating these findings into clinical practice will require well-designed interventions targeting maternal nutrition, breastfeeding support, and microbiota-modulating strategies. Although still exploratory, these approaches may help support early microbial colonization and improve long-term health trajectories.

## 9 Conclusion

Maternal milk mediates microbial transfer to infants through integrated pathways, including the entero-mammary route, nipple and areolar skin, mammary ducts, and retrograde flow from the infant’s oral cavity. These processes are shaped by maternal characteristics, delivery mode, lactation stage, infant-related factors, probiotic or antibiotic exposure, and environmental influences.

Through these transmission routes, the milk microbiota contributes to the establishment of infant gut, oral, and respiratory microbial communities, promoting colonization by beneficial taxa such as *Bifidobacterium* and *Lactobacillus* while constraining potential pathogens. These early microbial interactions support immune maturation, metabolic programming, and long-term protection against allergic, inflammatory, and metabolic disorders. Milk bioactive components-including oligosaccharides, lipids, polyamines, and micronutrients-further create selective ecological niches that enhance microbial growth, function, and host-microbe interactions.

Breastfeeding remains a cornerstone strategy for optimizing infant microbiome development, particularly in high-risk contexts such as cesarean delivery, prematurity, maternal dysbiosis, or early life microbial disruption. Milk bioactives may further enhance probiotic efficacy by supporting microbial growth, gut colonization, and immune modulation, even without substantial changes in overall milk microbial composition. Maternal probiotic supplementation during pregnancy and lactation can enrich milk with beneficial bacteria, increase secretory IgA, modulate cytokines such as TGF-β, alter HMO profiles, and reduce inflammatory markers. When combined with infant probiotic supplementation, these maternal effects may exert synergistic benefits on early life microbial colonization and immune development.

In summary, the human milk microbiota represents a central contributor to early life microbial colonization and infant health. Elucidating its origins, determinants, and functional roles will be essential for developing targeted strategies to optimize maternal-infant microbial transfer, support healthy growth, and promote lifelong health. Together, current evidence indicates that the human milk microbiota constitutes a dynamic ecological system whose origins and determinants collectively shape maternal-infant microbial transfer and early life microbiome assembly.
